# Dihydrolipoamide dehydrogenase (DLD) is a novel molecular target of bortezomib

**DOI:** 10.1038/s41419-024-06982-2

**Published:** 2024-08-13

**Authors:** Yu Feng, Hongmei Luo, Jingcao Huang, Yue Zhang, Jingjing Wen, Linfeng Li, Ziyue Mi, Qianwen Gao, Siyao He, Xiang Liu, Xinyu Zhai, Xin Wang, Li Zhang, Ting Niu, Yuhuan Zheng

**Affiliations:** 1 Department of Hematology/Institute of Hematology, West China Hospital, Sichuan University, Chengdu, China; 2 Department of Hematology, Mianyang Central Hospital, School of Medicine, University of Electronic Science and Technology of China, Mianyang, China; 3 School of Life Science, Sichuan University, Chengdu, China

**Keywords:** Myeloma, Cancer metabolism

## Abstract

Proteasome inhibitors (PIs), such as bortezomib and calfizomib, were backbone agents in the treatment of multiple myeloma (MM). In this study, we investigated bortezomib interactors in MM cells and identified dihydrolipoamide dehydrogenase (DLD) as a molecular target of bortezomib. DLD catalyzes the oxidation of dihydrolipoamide to form lipoamide, a reaction that also generates NADH. Our data showed that bortezomib bound to DLD and inhibited DLD’s enzymatic function in MM cells. DLD knocked down MM cells (DLD-KD) had decreased levels of NADH. Reduced NADH suppressed assembly of proteasome complex in cells. As a result, DLD-KD MM cells had decreased basal-level proteasome activity and were more sensitive to bortezomib. Since PIs were used in many anti-MM regimens in clinics, we found that high expression of DLD correlated with inferior prognosis of MM. Considering the regulatory role of DLD in proteasome assembly, we evaluated DLD targeting therapy in MM cells. DLD inhibitor CPI-613 showed a synergistic anti-MM effect with bortezomib in vitro and in vivo. Overall, our findings elucidated DLD as an alternative molecular target of bortezomib in MM. DLD-targeting might increase MM sensitivity to PIs.

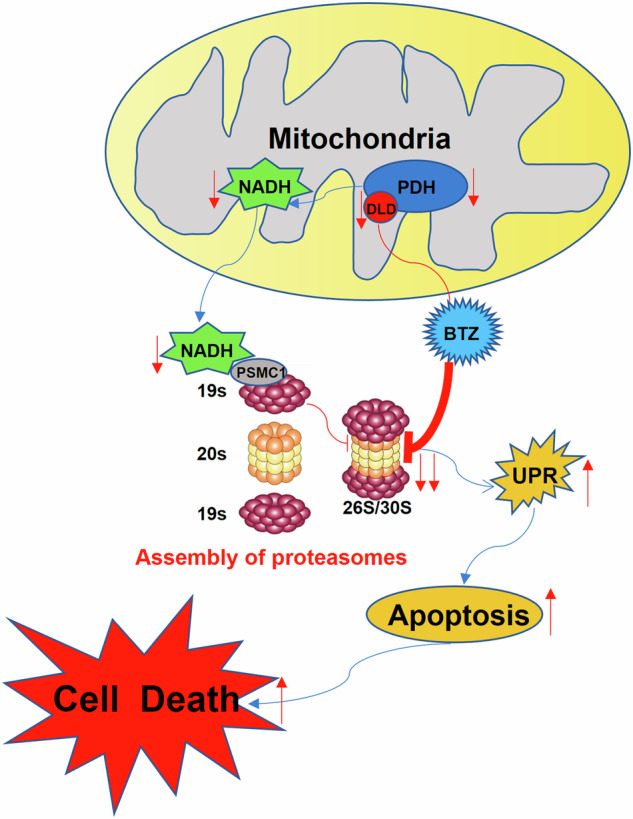

## Introduction

Multiple myeloma (MM), a plasma cell cancer in bone marrow, is highly prevalent in the elderly and remains incurable [1, 2]. In the past few decades, the application of proteasome inhibitors (PIs), such as bortezomib and calfizomib, has significantly improved the prognosis of MM [3]. However, primary or secondary bortezomib resistance is very common in clinical practice [4]. PI-resistance involves complicated mechanisms and still awaits further investigations.

Dihydrolipoamide dehydrogenase (DLD) is a flavin-dependent enzyme in the inner membrane of mitochondria. DLD functions as the E3 component of the complex of pyruvate dehydrogenase (PDH), the α-ketoglutarate dehydrogenase, and the branched-chain α-ketone dehydrogenase [5]. Among these three enzymes, the PDH complex is a rate-limiting enzyme that connects glycolysis to the tricarboxylic acid cycle. It consists of E1 (including catalytic α [PDHA] and regulatory β [PDPB] subunits), E2 (dihydroacyl transacetylase), E3 (DLD), and PDH complex component X (PDHX) as an E3 binding protein [6]. Although PDHX does not have a catalytic function, it is necessary for maintaining the activity of PDH enzyme complexes [7]. In all the above reactions that DLD catalyzed, niacinamide adenine dinucleotide, which is divided into an oxidized state (NAD^+^) and a reduced state (NADH), is an important coenzyme for electron transfer. The NADH can release two electrons during conversion to NAD^+^ [8]. Therefore, DLD is a redox enzyme that plays a crucial role in energy metabolism and redox balance [9]. The role of DLD in MM is not elucidated. Only one literature established and validated a multi-gene prognostic model including the DLD gene in MM through bioinformatics methods [10]. In this study, we performed a biotinylated pull-down assay using biotin-conjugated bortezomib (Bio-BTZ) and MM cell lysate. Proteins pulled down were analyzed by mass spectrometry. According to our result, DLD was a bortezomib-binding protein.

## Materials and methods

### Patient samples and patient omics data

The study was approved by the Ethics Committee of West China Hospital of Sichuan University (protocol no.114). West China Hospital of Sichuan University provided BM aspiration and biopsy for newly diagnosed MM patients. CD138^+^ cells, as mentioned earlier [11], were isolated primitive MM cells from BM. The gene expression profile (GEP) dataset and clinical data of MM patients from the MMRF CoMMpass study were obtained from the MMRF official website (https://research.themmrf.org). The MM GEP dataset GSE9782-GPL96, GSE26760, GSE5900, and GSE13591 were obtained from the NCBI GEO database (https://www.ncbi.nlm.nih.gov/geo), the corresponding clinical data was downloaded from the Oncome database (https://www.oncomine.org).

### Reagents and antibodies

Bortezomib (catalog S1013), Carfilzomib (CFZ) (catalog S2853), Melphalan (MEL) (catalog S8266), Dexamethasone (DEX) (catalog S1322), Doxorubicin (DOX) (catalog S1208), and Selinexor (SEL) (catalog S7252) were purchased from Selleck Chemicals (TX, USA). The recombinant human DLD, PDHX, PSMB5, PSMC1, and ΔGxGxxG PSMC1 Mutant proteins were customized by DetaiBio (Nanjing, China). Western blot antibodies against PSMB5 (catalog sc-393931), CHOP (catalog sc-7351), 20S Proteasome α1/α2/α3/α5/α6/α7 (catalog sc-58412), and eIF2a (catalog sc-133132) were purchased from Santa Cruz Biotechnology (CA, USA). Western blot antibodies for DLD (catalog 16431-1AP), PSMC1 (catalog 11196-1-AP), PSMA4 (catalog 11943-2-AP), p-eIF2α (Ser51) (catalog 28740-1-AP), p-PERK (Thr982) (catalog 82534-1-RR), ATF6 (catalog 24169-1-AP), PDHX (Cat#10951-1-AP), and β-actin (catalog 66009-1-Ig) were purchased from Proteintech (HuBei, China). Antibodies against ATF4 (catalog 11815), cleaved caspase3 (catalog 9661), and XBP-1s (catalog 83418) were derived from cell signaling technology (MA, USA). A protein blotting antibody targeting PERK (catalog HA500326) was obtained from Huabio (Zhejiang, China).

### Cells and cell culture

Human MM cell lines AMO.1, IM9, LP1, ARD, and KMS11 were cultured in RPMI-1640 medium (HyClone, catalog SH30809.01, UT, USA) with the addition of 10% fetal bovine serum (Excel, catalog FCS500, Shanghai, China) at 37 °C and 5% CO_2_. All the cell lines were subjected to short tandem repeat analysis and tested for contamination of mycoplasma before use. In order to establish DLD knocked down (DLD-KD) MM cells and CTR-KD cell lines, the pLKO.1 control vector (MilliporeSigma, catalog SHC002) and pLKO.1 plasmid with human DLD shRNA (5’- GCAAATCTTGCTGCGTCATTT-3’) was respectively, used to produce CTR-KD and DLD-KD viruses. The lentiviral particles were produced by transfection into HEK293T cells (ATCC) as described earlier [12]. MM cells infected with lentivirus were cultured in a medium with 1 μg/mL puromycin until stable knock-down cell lines were obtained.

### Biotin pull-down analysis

The Bio-BTZ was synthesized according to an unpublished protocol. The magnetic beads coated streptavidin were from Thermo Fisher Scientific (Dynabeads MyOne Streptavidin T1, catalog 65602, MA, USA). Bio-BTZ was added to the cell lysate (1 mg/mL, 500 μL PBS) or recombinant protein (1 μg/mL, 500 μL PBS), gently rotated at 4 °C for 2 h, then magnetic beads (100 μL per sample) were added, they were kept rotating overnight at 4 °C. On the second day, the magnetic beads were washed 4 times with ice PBS, and the bound protein was released by adding a two-dimensional electrophoresis loading buffer. Biotin was used as a negative control.

### Two-dimensional electrophoresis and protein silver staining

The aforementioned pull-down products were separated by the first phase isoelectric focusing electrophoresis and the second phase sodium dodecyl sulfate-polyacrylamide gel electrophoresis (SDS-PAGE) as described earlier [13], and then the SDS-PAGE gels were performed by a Protein Silver Staining Kit (Beyotime, catalog 65602 P0017S, Shanghai, China).

### Mass spectrometry identification of proteins in gel dots

After comparing the protein silver staining patterns of Bio-BTZ and Biotin, the differential gel dots in the two groups were cut off and identified by mass spectrometry by Nano Quadrupole LTQ Orbitrap Fusion Lumis Tribrid (Thermo, Orbitrap fusion Lumos) as previously described [14].

### Microscale thermophoresis (MST)

Recombinant proteins were labeled with fluorescence by Monolith His-Tag Labeling Kit RED-tris-NTA 2nd Generation (Nano Temper, MO-L018, Beijing, China) and mixed with small molecule compounds for MST detection (Monolith X, Bavaria, Germany) as previously described [15].

### Cell viability and apoptosis assay

Cell Count Kit-8 (CCK-8) (Oriscience, catalog CB101-10 mL, Sichuan, China) was used to test cell viability. Cells (2 × 10^4^ cells per well) were inoculated into a 96-well plate and tested after drug exposure. Cells were incubated with CCK-8 for 1 h and absorbance was measured at 450 nm by a microplate luminometer (Molecular Devices, CA, USA).

For cell apoptosis measurement, the MM cells were stained with the Annexin V-FITC/PI assay kit (4A Biotech, catalog FXP018-100, Beijing, China). Then, the cells were detected by flow cytometry (Beckman Coulter, CA, USA).

### Sucrose density gradient centrifugation

The sucrose density gradient with a continuous variation of 10–40% was generated by a density gradient preparation device (BIOCOMP, CA), and the cell lysates of CTR-KD and DLD-KD were added to the top layer, then they were placed in an ultra-high-speed centrifuge (Beckman coulter, CA, USA) at 268,000 g and 4 °C for 3 h. Finally, the substance after centrifugation was separated into 15 EP tubes by a density gradient separation system (BIOCOMP, CA).

### Proteasome activity assay

The protein content in cell lysates was quantified by the BCA assay (catalog CW0014S, CWBIO, Jiangsu, China). An equal amount of cell extract (80 μg) was added to a white 96-well plate, and the proteasome buffer (50 mM Tris, pH 7.5, 5 mM MgCl_2_, 10% glycerol, 1 mM ATP, 1 mM DTT) was added to a final volume of l00 μL. Subsequently, 100 μL of Suc-LLVY-AMC (20 nM) was loaded into each reaction. The above steps were performed on ice. Then, the reaction mixture was incubated at 37 °C for 90 min and the fluorescence intensity at 450 nm was measured by a fluorescence microplate reader (Molecular Devices, CA, USA).

### PDH activity detection

Detection of PDH enzyme activity in cells by a reagent kit (Abcam, catalog ab109902, MA, USA).

### NADH detection in cells

The concentration of NADH in cells was detected by NAD^+^/NADH detection kit (Beyotime, catalog S0175, Shanghai, China) as described in the manual.

### Immunohistochemical staining (IHC)

Paraffin sections were used for IHC as previously described [16]. The dilution ratio of antibodies for cleaved caspase3 and DLD used in IHC was 1:200.

### Animal studies

Animal work was approved by the Animal Ethics Committee of West China Hospital of Sichuan University (protocol no. 20240226004). The human MM xenograft model was established as follows, the human MM cell line which expressed luciferase (ARD-luc: Ct-KD or Dl-KD) was injected into a female 8-week-old severely immunodeficient NOD Prkdcscid IL2rgtm1/Bcgen (NCG) mice (Biocytogen, Beijing, China) via tail vein (2 × 10^6^ per mouse). Bioluminescence Imaging (IVIS Spectrum, PerkinElmer, MA, USA) was used to evaluate tumor burden in mice. Tumor-bearing mice were randomly divided into four groups, with five mice in each group. They received PBS (control group) or BTZ (0.5 mg/kg, twice a week intravenous injection). We conducted weekly bioluminescence imaging to check the tumor burden in mice. Each group of mice received a 3-week treatment. The tumor burden of mice was assessed through bioluminescence imaging and detection of kappa light chain content (Abcam, catalog ab157709, MA, USA) in the peripheral blood serum of mice. The therapeutic effect and prognosis of mice were evaluated by survival curves. When the mice reached the preset endpoint of paralysis, drowsiness, or weight loss exceeding 20%, they were euthanized with a CO_2_ tank.

In our animal experiment to investigate the synergistic effect of CPI-613 and BTZ in vivo, we constructed an MM animal model by injecting ARD-luc cells into the tail vein of the immunodeficient NCG mice (2 × 10^6^ per mouse). Subsequently, the tumor-bearing mice were randomly divided into four groups, with six mice in each group. These four groups were treated with PBS, BTZ (0.5 mg/kg, twice a week), CPI-613 (25 mg/kg, three times a week), and BTZ combined with CPI-613. After the 7th day of treatment, peripheral blood samples were collected from each group of mice for blood routine and liver and kidney function testing, one mouse in each group was euthanized, and the heart, liver, lungs, and kidneys of the mice were collected and made into paraffin sections for hematoxylin-eosin (HE) staining to evaluate the organ side effects of the drugs. Other matters were similar to the animal experiments mentioned above.

### Statistical analyses

The data was analyzed using GraphPad Prism 8.0.2 software. The significance between the two groups was tested by Student’s *t*-test. One-way ANOVA was used to estimate differences between three or more groups. Two-way ANOVA was used to compare the differences in peripheral blood light chains over time among different treatment groups of mice, the differences in different treatment concentrations or times of CPI-613, and the differences between different concentration combinations of CPI-613 and BTZ. The log-rank (Mantel-Cox) test was used to analyze the survival rates of patients and tumor-bearing mice. The results were displayed as mean ± standard deviation. *P* < 0.05 was considered statistically significant.

## Results

### Bortezomib binding to DLD

**Fig. 1:**
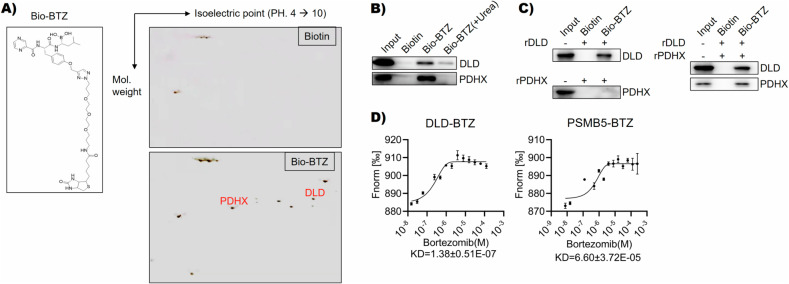
Identification of DLD as a bortezomib (BTZ) binding protein. **A** The image on the left was the structural diagram of BTZ connected to Bio-BTZ. The image on the right was the protein silver staining diagram of two-dimensional electrophoresis gel (biotin on the top and Bio-BTZ on the bottom). After comparison between the top and the bottom, the mass spectrum identification results of different gel spots were shown at the bottom, and DLD and PDHX were preliminarily screened. **B** The western blot results of the biotin pull-down product showed that Bio-BTZ could pull down DLD and PDHX, but after adding urea, Bio-BTZ could only pull down DLD, which indicated that only DLD could bind to Bio-BTZ. **C** The biotin pull-down experiment of recombinant proteins (DLD or PDHX) showed that when DLD and PDHX were present simultaneously, they could be pulled down by Bio-BTZ, when DLD was present alone, it could also be pulled down by Bio-BTZ, however, when only PDHX was present alone, PDHX could not be pulled down by Bio-BTZ. **D** The left image showed that DLD could bind to BTZ, the KD value was 1.38 ± 0.51 E-7. The right image showed a positive control.

The Bio-BTZ was synthesized and validated as previously described [17]. To identify molecular targets of bortezomib (BTZ), we performed a biotinylated pull-down assay using MM cell lysate. The pulled-down proteins were separated by two-dimensional electrophoresis, followed by silver staining for protein visualization. Unique staining spots in Bio-BTZ pulled-down samples were cut off and analyzed by mass spectrometry. As shown in Fig. 1A and Supplementary Table 1, we identified DLD and PDHX, both of which belonged to a mitochondria enzyme complex. Biotinylated pull-down assay followed by western blot confirmed that BTZ could bind to DLD even under the stringent denature condition with urea (Fig. 1B). PDHX could not be pulled down under urea conditions, a result might indicate that the interaction between PDHX and BTZ was not as strong as that of DLD and BTZ. Biotinylated pull-down assay using recombinant proteins showed that BTZ bound directly to DLD, but not PDHX (Fig. 1C). Since DLD and PDHX had protein-protein interactions and formed complexes in cells, Bio-BTZ could pull down PDHX in the presence of DLD (Fig. 1C). The interaction of BTZ and DLD was further confirmed by MST technology (Fig. 1D). Of note, MST examined the original form of BTZ instead of biotinylated BTZ in binding with DLD. According to the MST, the binding affinity of BTZ to DLD and to PSMB5, which was a subunit of proteasome complex and the functional target of BTZ, was comparative. In addition, the Biotin pull-down assay suggested that DLD competed with PSMB5 in BTZ binding (Fig. S1A). In summary, our findings showed that BTZ was bound to DLD in MM cells.

### DLD expression in MM

**Fig. 2:**
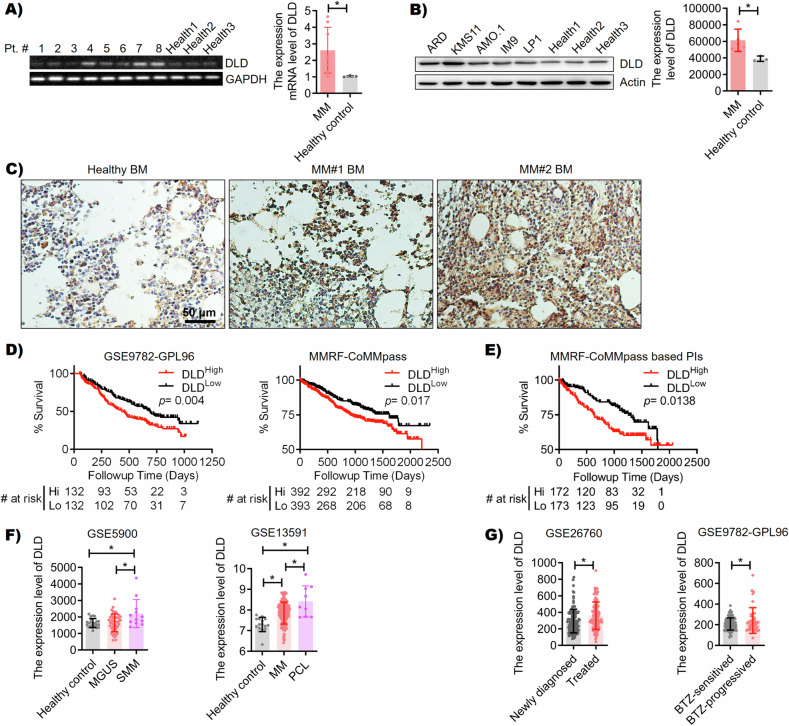
DLD expression in myeloma. **A** The electrophoresis patterns of CD138^+^ cells in the BM of 8 newly diagnosed MM patients and three healthy controls by RT-qPCR showed the mRNA expression level of DLD in newly diagnosed MM patients was significantly higher than that in healthy controls (*p* < 0.05). **B** ARD, KMS11, AMO.1, IM9, and LP1 all expressed DLD and were significantly higher than healthy controls (*p* < 0.05). **C** The expression levels of BM DLD protein in one healthy control and 2 MM patients were 9.99%, 16.86%, and 21.22%. **D** In GSE9782-GPL96 (left, ntotal = 264, *p* = 0.004) and MMRF CoMMpass (right, ntotal = 785, *p* = 0.017), survival analysis showed that patients with high expression of DLD had a poor prognosis. **E** In patients treated based on PIs from MMRF CoMMpass, survival analysis also showed that patients with high expression of DLD had poor prognoses (ntotal = 345, *p* = 0.0138). **F** In GSE5900, smoldering MM (sMM) patients (*n* = 12) had higher DLD expression than monoclonal gammopathy of undetermined significance (MGUS) patients (*n* = 44, *p* = 0.0072) or healthy controls (*n* = 22, *p* = 0.0062). In GSE13591, the expression of DLD was higher in MM patients (*n* = 133, *p* = 0.0001) than in healthy controls (*n* = 15), the expression of DLD was highest in plasma cell leukemia patients (*n* = 9, *p* < 0.0001). **G** In GSE26760, the patients receiving secondary treatment (*n* = 118, *p* = 0.0015) had higher expression of DLD compared to newly diagnosed patients (*n* = 122). In GSE9782-GPL96, compared to patients who are sensitive to BTZ treatment (*n* = 85), patients with disease progression after BTZ treatment (*n* = 41, *p* = 0.042) have higher levels of DLD expression, *p < 0.05.

We detected the mRNA expression of DLD in primary MM cells isolated from patients, and CD19^+^ B cells from three healthy individuals were used as controls, the quantitative results of reverse transcription-polymerase chain reaction (RT-qPCR) showed that the mRNA level of DLD in plasma cells of MM patients was higher than that of healthy controls (Fig. 2A). We also showed that human MM cell lines expressed DLD, similarly, CD19^+^ B cells from three healthy individuals served as controls, we quantitatively analyzed the results by ImageJ software, and the results showed that the protein expression of DLD in plasma cells of MM patients was higher than that of healthy controls (Fig. 2B). Immunohistochemistry results suggested that MM bone marrow (BM) had increased DLD expression, compared with that in healthy BM (Fig. 2C). Using publicly accessible datasets, we showed that MM patients with high DLD expression had inferior prognosis (Fig. 2D). In particular, the prognostic value of DLD expression was true in patients treated with PI-based regimens (Fig. 2E). In the same dataset, patients with del (13) or del (17) had higher DLD expression than those without such cytogenetic changes (Fig. S1B). Both del (13) and del (17) are considered high-risk cytogenetic changes in MM [18]. Furthermore, it seemed like DLD expression increased during MM progression (Fig. 2F). Compared with newly diagnosed patients, treated patients had high DLD expression in MM cells, and high DLD expression was observed in patients with progressive disease after BTZ treatment (Fig. 2G). Overall, our findings suggested that high DLD expression in MM correlated with BTZ-resistance and inferior prognosis.

### DLD regulates myeloma sensitivity to PIs

**Fig. 3:**
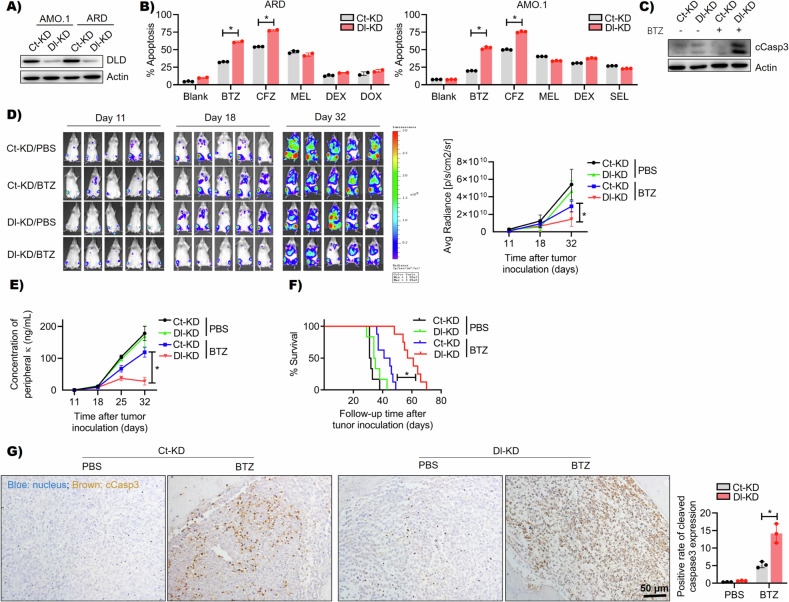
DLD regulates myeloma sensitivity to PIs. **A** Western blot showed knockdown of DLD in AMO.1 and ARD cells. **B** The results of flow cytometry combined with PI and Annexin V double staining also showed that after knocking down DLD, AMO.1, and ARD cells were only more sensitive to BTZ or CFZ (BTZ 5 nM 24 h, CFZ 4 nM 24 h, MEL 40 μM 24 h, DOX 0.5 μM 24 h, DEX 70 μM 48 h, SEL 0.5 μM 48 h) (*p* < 0.05). **C** Western blot showed that after BTZ treatment (5 nM 6 h), the expression of cleaved caspase3 was upregulated in DLD-KD cells. **D** Bioluminescence images of four groups (Ct-KD/Blank, Ct-KD/BTZ, Dl-KD/Blank, and Dl-KD/BTZ) of mice on the 11th, 18th, and 32nd days. The results showed that compared to Ct-KD, the Dl-KD group of mice had better BTZ treatment effects (*p* < 0.05). **E** The κ light chain results of peripheral blood from four groups of mice on the 11th, 18th, 25th, and 32nd days also showed that the Dl-KD/BTZ group had a lower level of κ light chain than Ct-KD/BTZ (*p* < 0.05). **F** Survival curves from four groups of mice showed that after receiving BTZ treatment, the survival time of the Dl-KD group was significantly longer than that of the Ct-KD group (*p* < 0.05). **G** The IHC results of pathological sections of leg bones from four groups of mice showed that BTZ therapy induced higher expression of cleaved caspase3 in the Dl-KD tumor bed than Ct-KD (brown color showed positive staining for cleaved caspase3, *p* < 0.05), *p < 0.05.

To examine the function of DLD in MM chemoresistance, we generated DLD-KD MM cell lines using the DLD shRNA virus (Fig. 3A). Compared with CTR-KD MM cells, DLD-KD cells had increased apoptosis when the cells were treated with PIs, such as BTZ and CFZ. However, DLD did not affect the drug sensitivity of MM to MEL, DEX, DOX, or SEL (Fig. 3B, Figs. S1C and S2). Western blot further confirmed that BTZ induced caspase3 cleavage in DLD-KD cells (Fig. 3C). Next, we established a human MM cell line xenograft mouse model in immunodeficient mice, after tumor-bearing in mice, we confirmed the knockdown of DLD in mice by IHC analysis (Fig. S3A). After the tumor establishment, the animals were treated with peritoneal injection of BTZ (0.5 mg/kg, twice a week) or PBS. IVIS assay showed the tumor burdens in vivo (Fig. 3D). According to the luciferase intensity and circulating M protein level, shown in Fig. 3D, E, respectively, DLD-KD MM was more sensitive to BTZ treatment in vivo than CTR-KD MM. The mice bearing DLD-KD MM treated with BTZ had prolonged survival (Fig. 3F). According to an immunohistochemistry assay using tumor biopsies in animal experiments, BTZ induced significant tumor cell apoptosis, measured by caspase three cleavage, in DLD-KD MM (Fig. 3G). Overall, our data suggested that DLD regulated MM cells’ chemoresistance to PIs: DLD^Low^ MM cells were more sensitive to PIs.

### DLD regulates proteasome complex assembly in myeloma cells

**Fig. 4:**
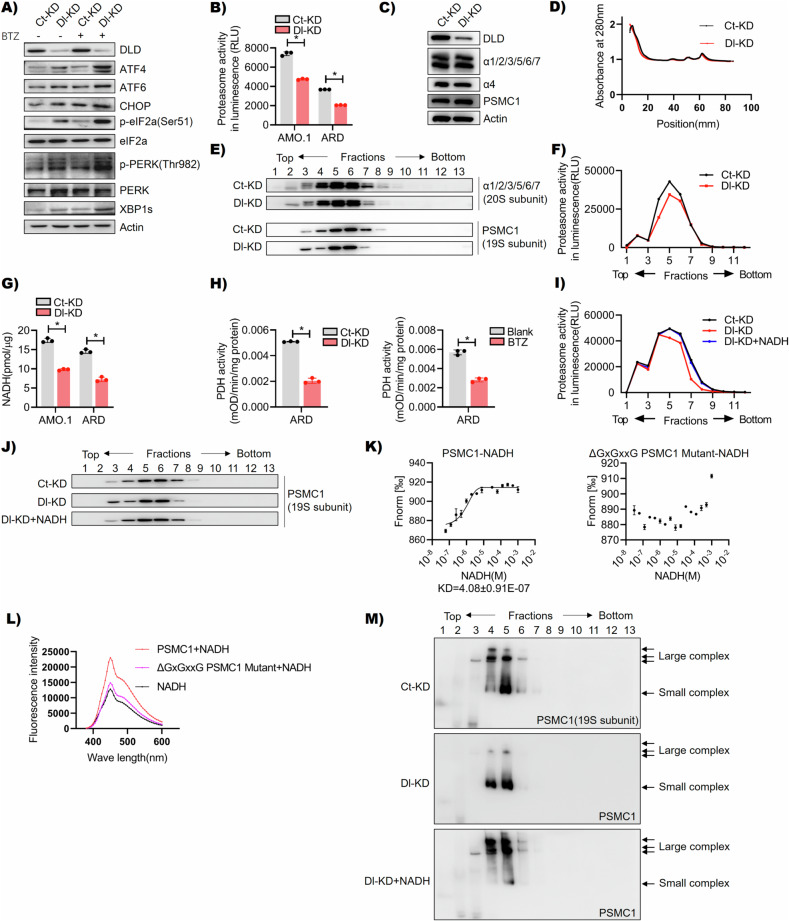
DLD regulates proteasome complex assembly in myeloma cells. **A** The western blot results showed that compared with Ct-KD cells, the expression level of ATF4, ATF6, CHOP, p-eIF2α, p-PERK, and XBP1s from Dl-KD cells was upregulated, and after BTZ treatment, the above proteins were more significantly upregulated in Dl-KD cells. **B** Proteasome activity detection showed that compared to Ct-KD, the Dl-KD group showed a decrease in proteasome activity in the AMO.1 or ARD cell line (*p* < 0.05). **C** The western blot results showed no significant difference in the expression levels of proteasome subunits (α1/2/3/5/6/7, α4, and PSMC1) between Ct-KD and Dl-KD cells. **D** The separated components after sucrose density gradient centrifugation were subjected to the western blot. The results showed the protein quantification during sucrose density gradient separation displayed that there was no significant difference in protein content between the separated components of Ct-KD and Dl-KD cell lysates. **E** The western blot results from (**D**) showed that compared with Ct-KD, the free 19S proteasome subunit of Dl-KD was increased, the complete proteasome was reduced. **F** The components in each tube from (**D**) were measured for proteasome activity by Suc-LLVY-AMC fluorescent substrates. The results showed that the proteasome activity of Dl-KD cells decreased in some fractions, compared to CTR-KD cells. **G** The NADH content detection showed that compared to Ct-KD, the NADH content in the Dl-KD group decreased in the AMO.1 or ARD cell line (*p* < 0.05). **H** The PDH activity of the Dl-KD group was lower than that of the Ct-KD group in ARD cells (the left image). In addition, we tested the PDH activity of ARD cells treated with BTZ (5 nM) for 30 min, and the results showed that the PDH activity of cells decreased after BTZ treatment (the right image). **I**, **J** The same method as (**D**–**F**) was used to demonstrate that adding NADH to rescue DLD knock-down induced inhibition of proteasome activity. **K** The results showed that NADH can interact with PSMC1, instead of ΔGxGxxG-PSMC1-Mutant, the KD value was 4.08 ± 0.91E-7. **L** The results showed that the NADH with recombinant protein PSMC1 instead of ΔGxGxxG PSMC1 Mutant showed significant enhancement at 450 nm. **M** Sucrose gradient analysis, natural gel electrophoresis, and the western blot showed that the addition of NADH promoted the assembly of PSMC1 in the DLD-KD MM cell macromolecular complex (30S), *p < 0.05.

Based on the above findings, DLD regulated MM cells’ chemoresistance to PIs, but not the resistance to other anti-MM agents. Since PIs induced MM cell death by activating unfolded protein response (UPR) cell signaling [19, 20], we examined UPR signaling molecules in CTR-KD versus DLD-KD MM cells. As shown in Fig. 4A, BTZ-treated DLD-KD cells had higher levels of UPR signaling activation than the drug-treated CTR-KD cells [21]. We also noticed that even under normal culture conditions without the drug treatment, DLD-KD cells had higher levels of UPR signaling molecule expression, such as ATF4, ATF6, CHOP, p-eIF2α(Ser51), p-PERK(Thr982), and XBP1s, than CTR-KD cells. Therefore, we hypothesized that DLD knocking down might affect the ubiquitin-proteasome system and UPR cell signaling. Proteasome activity measurement confirmed that even without PI treatment, DLD-KD cells had decreased proteasome activity (Fig. 4B). The proteasome is a macromolecule complex in cells. The 26S proteasome is a large molecular complex composed of one 20S catalytic core and one 19S regulatory complex, while the 30S proteasome is composed of one 20S catalytic core and two 19S regulatory complexes. Furthermore, each 20S catalytic core and 19S regulatory complex are composed of multiple different protein subunits [20]. We found that the total level of selected proteasome subunits was not changed after DLD knocking down (Fig. 4C). Next, we fractionated cellular components by sucrose gradient centrifugation, which separated cellular macromolecules according to their sizes and structures [22]. Fractionized cell components were analyzed by SDS-PAGE. The result showed that DLD-KD cells had different proteasome subunits, such as PSMC1, distribution in sucrose gradient fractions, while the total protein level in each fraction remained similar in CTR-KD versus DLD-KD (Fig. 4D, E). Such findings might indicate the assembly of proteasome complex was impaired in DLD-KD cells. We also examined proteasome activity associated with each sucrose gradient fraction and found that DLD-KD cells had reduced proteasome activity in some fractions, such as in fractions 3–7 (Fig. 4F). Next, we treated CTR-KD and DLD-KD cells with BTZ, and further validated that BTZ could regulate the assembly of proteasomes (Figs S3B, S3C). As described earlier, DLD-catalyzed reactions also generated NADH [8]. According to the literature, NADH could enhance proteasome activity by binding to PSMC1 [23]. We found that DLD-KD MM cells had reduced intracellular NADH (Fig. 4G). In addition, we found a significant decrease in PDH enzyme activity in MM cells treated with BTZ (Fig. 4H). Important, the addition of NADH rescued DLD knocking down-induced repression of proteasome activity (Fig. 4I). According to the result of the sucrose gradient assay followed by SDS-PAGE, the addition of NADH recovered DLD knocking down induced proteasome assembly impairment (Fig. 4J). MST assay confirmed the binding of PSMC1 and NADH (Fig. 4K). The PSMC1 mutant (ΔGxGxxG-PSMC1) with rupture of NADH binding site had negative results in the MST assay. Furthermore, according to a previous study, the unbound NADH excited at 380 nm had a maximum emission of fluorescence at 450 nm, and the binding of NADH to proteins enhanced NADH-related fluorescence intensity [24]. Thus, we conducted a similar assay to show NADH binding to PMSC1 by fluorescence intensity (Fig. 4L). The results of Fig. 4K, L were consistent with previous findings reported by Tsvetkov et al. [23] last but not least, sucrose gradient assay followed by native gel electrophoresis and western blot showed that NADH addition promoted PSMC1 assembly in macromolecule complex (30S) in DLD-KD MM cells (Fig. 4M). Overall, we demonstrated the mechanism of DLD knocking down induced MM hyper-sensitivity to PIs. DLD^Low^ MM cells had repressed PDH enzyme activity and reduced endogenous NADH levels. Low NADH in cells inhibited the formation of proteasome complexes.

### DLD-targeting therapy in myeloma

**Fig. 5:**
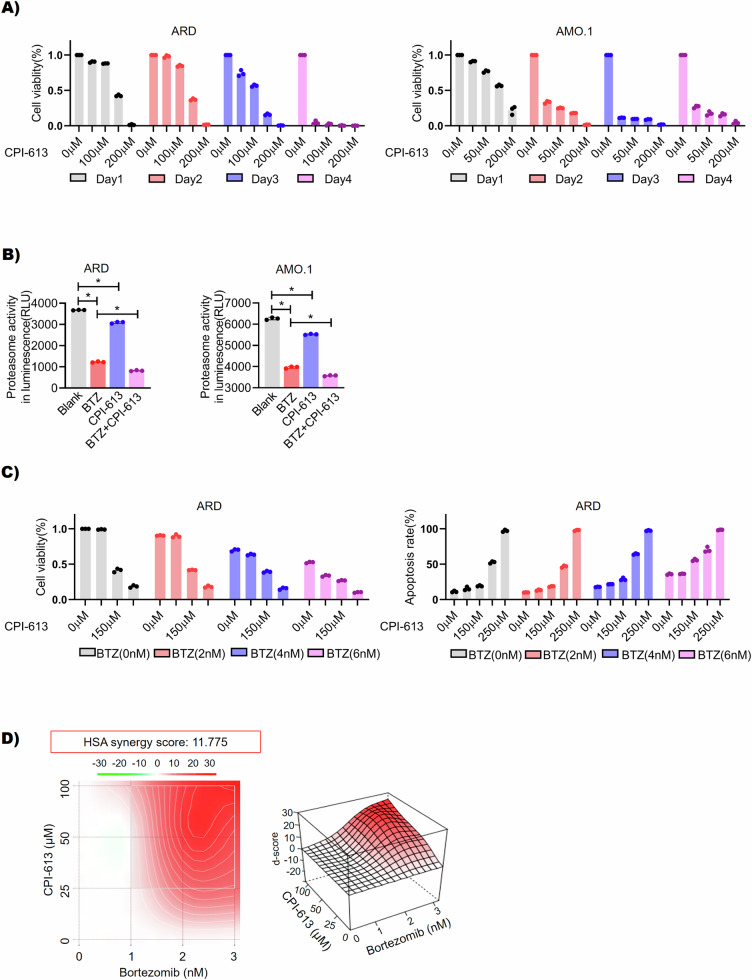
DLD targeting therapy in myeloma in vitro. **A** The time and concentration dependence of CPI-613 in MM cell lines (ARD and AMO.1) was detected by CCK-8 (CPI-613 25 μM, 50 μM, 100 μM, 150 μM, and 200 μM, *p* < 0.05). **B** After treating AMO.1 or ARD cells with BTZ monotherapy, CPI-613 monotherapy, or both drugs simultaneously, the proteasome activity of the cells was detected. The results showed that the proteasome activity of BTZ monotherapy and CPI-613 monotherapy groups both decreased and the group treated with BTZ combined with CPI-613 showed the greatest decrease in proteasome activity (*p* < 0.05). **C** ARD cells were treated with CPI-613 and various concentrations of BTZ (alone or in combination). CCK-8 was used to detect cell viability (as shown in the left figure, *p* < 0.05), and cell apoptosis was detected by flow cytometry with an apoptosis assay kit (as shown in the right figure, *p* < 0.05). **D** The three-dimensional synergy diagram of CPI-613 and BTZ was shown in the figure, and the synergistic score (11.775) of CPI-613 and BTZ in ARD cells was calculated by SynergyFinder based on the data shown in (**C**), *p < 0.05.

As shown above, DLD was a molecular target of BTZ and correlated with PI sensitivity in MM cells. Next, we evaluated DLD-targeting therapy in MM treatment. CPI-613 was a fatty acid analog that inhibited DLD activity, blocked mitochondrial energy metabolism in tumor cells, and induced cell apoptosis [25]. CPI-613 has been evaluated in a phase I clinical trial for the treatment of hematological cancers (NCT03504410) [26]. However, the anti-MM activity of CPI-613 has not yet been examined. Here we showed that the CPI-613 single agent exerted potent anti-MM activity in time-dependent and dose-dependent manners [27] (Fig. 5A). The addition of a CPI-613 single agent slightly inhibited proteasome activity in MM cells, while the combination of BTZ and CPI-613 strongly repressed proteasome activity (Fig. 5B). Next, we evaluated the combination dosages of BTZ and CPI-613 in vitro. Our result suggested that BTZ and CPI-613 exhibited synergistic anti-MM cytotoxicity in vitro [19] (Fig. 5C, D and Fig. S4).

**Fig. 6:**
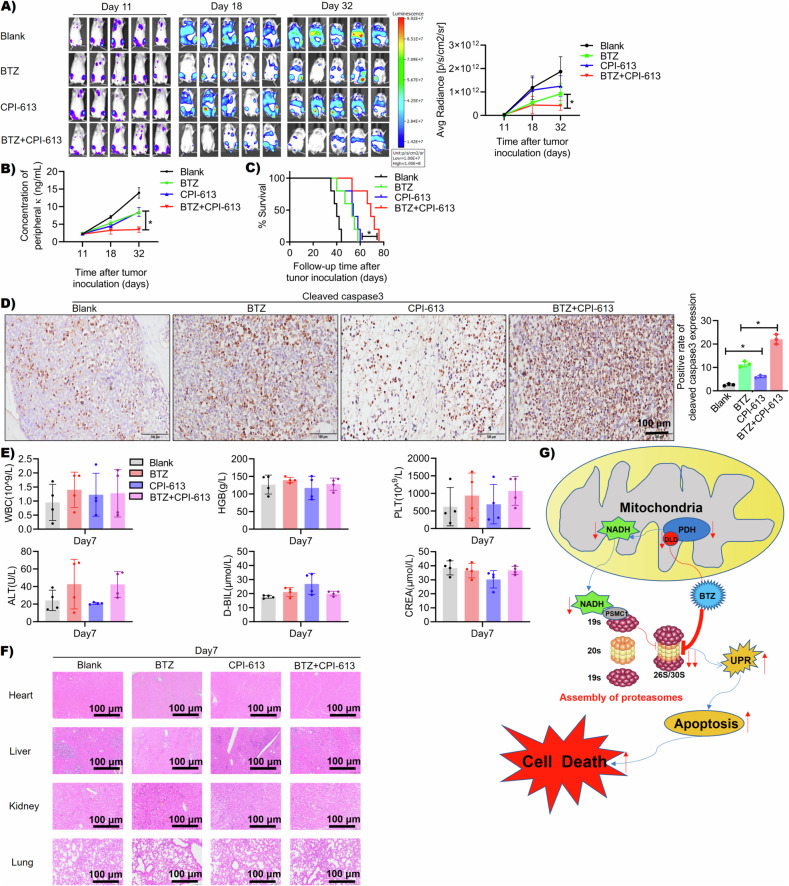
DLD targeting therapy in myeloma in vivo. **A** Bioluminescence image of four groups of mice (Blank, BTZ, CPI-613, and BTZ + CPI-613) on the 11th, 18th, and 32nd days. The results showed that the BTZ combined with the CPI-613 treatment group had the best therapeutic effect (*p* < 0.05). **B** The kappa light chain detection results on the peripheral blood of the four groups of mice on the 11th, 18th, and 32nd days showed that the BTZ combined with the CPI-613 treatment group had lower κ light chain content than the BTZ treatment group (*p* < 0.05). **C** The survival curves from four groups of mice showed that the BTZ combined with the CPI-613 treatment group had the longest survival time (*p* < 0.05). **D** The IHC staining results of leg bone pathological sections from four groups of mice showed that the BTZ combined with the CPI-613 group had the highest expression of cleaved caspase 3 (brown color showed positive staining for cleaved caspase 3, *p* < 0.05). **E** The results showed that there were no significant differences in white blood cells (WBC), hemoglobin (HGB), platelets (PLT), alanine aminotransferase (ALT), direct bilirubin (D-BIL), and creatinine (CREA) between the 7th day from each group of mice (*p* > 0.05). **F** The HE staining results of organ pathology sections of mice in each group on the 7th day after treatment showed that no significant pathological changes were found in the heart, liver, lungs, and kidney issues. **G** Pattern diagram of DLD regulating PIs resistance in MM. After the interaction between BTZ and DLD, the activity of its constituent enzyme PDH and the content of its catalytic product NADH both decreased, leading to a decrease in the binding of NADH to the 19S proteasome subunit PSMC1, resulting in abnormal assembly of proteasomes and further inhibition of proteasome activity. The decrease in proteasome activity activated the UPR signaling pathway, inducing cell apoptosis.

We also evaluated the efficacy of dual-drug in vivo in a human MM cell xenograft mouse model. The MM-bearing mice were treated with either PBS (Blank), BTZ (0.5 mg/kg, twice a week), CPI-613 (25 mg/kg, three times a week), or a combination of CPI-613 and BTZ. As shown in Fig. 6A–C, the dual-drug treatment resulted in a better outcome than either single agent. The dual-drug treatment induced increased tumor cell apoptosis, examined by the expression of cleaved caspase 3 in mouse BM biopsies (Fig. 6D). At last, we evaluated the in vivo toxicity of the dual-drug. Overall, the dual-drug treated mice had normal blood cell counts, and normal hematological and renal functions (Fig. 6E). In addition, there were no noteworthy pathological changes in heart, liver, lung, and kidney tissues from the dual-drug treated mice (Fig. 6F). In summary, our findings showed that DLD was a molecular target of BTZ. BTZ addition inhibited DLD-related enzymatic function and reduced intracellular NADH levels. Since NADH contributed to proteasome complex assembly, BTZ-DLD interaction promoted BTZ cytotoxicity in MM cells (Fig. 6G).

## Discussion

MM is a plasma cell cancer with hyperactive protein synthesis in cells. Therefore, MM had a notable ubiquitin-proteasome system to maintain the intra-cellular protein homeostasis. PIs exerted anti-MM activity through binding and inhibiting proteasome subunits, such as PSMB5, PSMB1, etc., and initiated UPR cell signaling that led to MM cell death. As revolutionary anti-MM compounds, PIs were now backbone agents in different MM treatment regimens [3]. BTZ was a small molecule, and it was reasonable to speculate that BTZ had multiple interactors in cells. Perbandt et al. showed that BTZ and CFZ bound to and inhibited β-lactamase [28]. A previous study from our group suggested that BTZ bound to ISG20L2, known as an exonuclease, in MM cells [17]. In this study, we conducted a proteomics-based screening of BTZ-binding proteins in MM cells using a biotinylation bortezomib compound. We found that in addition to proteasome-subunits targeting, BTZ also bound to DLD, which was a component of at least three enzyme complexes, PDH, the α-Ketoglutarate dehydrogenase, and the branched-chain α-ketone dehydrogenase [5]. In all the above enzymes, DLD catalyzed the hydrogenation reaction of NAD^+^ to NADH. Previous studies suggested that DLD was a determinant of cellular NADH [5]. The binding of BTZ to DLD was also confirmed by the MST method, in which the original form of BTZ was used. According to our data, BTZ exhibited comparable affinity in binding with PSMB5 or DLD. The binding of BTZ to DLD prompted us to investigate the correlation of DLD expression and MM sensitivity to BTZ. Our in vitro assays showed that DLD regulated MM sensitivity to PIs (BTZ and CFZ), but not other anti-MM agents including MEL, anthracycline agent DOX, or selective nuclear export inhibitor SEL, all of which had different mechanisms of action in targeting MM cells [29, 30]. Important, in a series of MM public datasets, DLD expression was significantly increased in relapsed MM patients who did not respond to BTZ. Such a result suggested a role of DLD in BTZ-responsive cell signaling in MM. Finally, our mechanistic study revealed that BTZ binding to DLD repressed NADH level in MM cells, and led to impairment of proteasome assembly. This finding was based on previous work done by Tsvetkov et al. [23]. Of note, low proteasome subunit expression in MM was associated with increased PI sensitivity [31]. In DLD^Low^-MM, since the proteasome assembly was affected and the functional proteasome complex level might be low, the BTZ exhibited superior treatment efficacy. PI resistance was a major obstacle in the MM clinic, and the underlying mechanisms were complicated and were still not fully demonstrated. Previous studies indicated that mutations [32] and changes in expression levels [33] of proteasome subunits, UPR signaling pathways [34], activation of autophagy [35], and even inhibition of apoptosis [36] could lead to tumor cell resistance to BTZ. Overall, our findings provided a novel mechanism of PI resistance in MM. We first linked the resistance of MM’s PIs to mitochondrial NADH metabolism.

The functions of DLD were diverse. In addition to its catalytic roles in mitochondrial enzyme complexes, DLD also had the following four moonlighting functions. First, DLD could promote the transport of galactose and maltose in *E. coli* [37]. Second, DLD monomers might have proteolytic activity [8]. Third, DLD had DNA binding activity, which might be involved in cell apoptosis regulation [38]. Fourth, DLD affected reactive oxygen species (ROS) generation. DLD catalyzed the generation of NADH, which in the subsequent electron transfer process combined with O_2_ to generate ROS [39]. Very few studies investigated DLD function in human cancers. In melanoma, vemurafenib reduced DLD expression in tumor cells and DLD was considered a promising target to overcome vemurafenib resistance [25]. Shin et al. showed that DLD-regulated cysteine deficiency induced ferroptosis in head and neck cancer [12]. Furthermore, Yoneyama et al. found that IgA autoantibodies against DLD could serve as a new diagnostic marker for endometrial cancer [40]. In this study, we demonstrated a new function of DLD in MM. We confirmed that DLD was a molecular target of BTZ, the interaction between BTZ and DLD promoted the anti-MM effect of BTZ.

Since the expression level of DLD could affect proteasome activity in MM cells, we investigated the efficacy of combination therapy using DLD inhibitors (CPI-613) and BTZ. CPI-613 and BTZ showed synergistic anti-MM activity in vitro and in vivo. CPI-613 mainly exerted pharmacological effects by inhibiting the activity of DLD [25]. It was of particular interest to us because this agent had been investigated in clinical trials. In the phase II clinical trial of CPI-613, researchers found that in relapsed or refractory acute myeloid leukemia (R/R AML) patients, the complete response rate was 52% (2000 mg/m^2^), with a median survival of 12.4 months in the first/second phase of combined treatment, CPI-613 in combination with cytarabine and mitoxantrone [26]. In another phase I clinical trial of CPI-613, which included 4 R/R MM patients, two patients remained stable after 2–4 cycles of CPI-613 treatment, while the other 2 R/R MM patients withdrew due to the disease progression [41]. Although CPI-613 single agent showed a limited anti-MM effect in patients, clinical trials suggested well-tolerance of the drug in patients. Based on our findings, a combination of CPI-613 and PIs might be promising in MM treatment.

### Supplementary information

Supplemental material

Supple. Table 1

Supplemental material

## Data Availability

The data can be obtained from the corresponding author upon reasonable request.
