# Supplementary material for: Dihydrolipoamide dehydrogenase (DLD) is a novel molecular target of bortezomib
Source: Cell Death Dis. 2024 Aug 13;15(8):588. doi: 10.1038/s41419-024-06982-2 (PMC11322525; doi:10.1038/s41419-024-06982-2)
Supplement: Supplementary file 1 — Supplemental material [file 41419_2024_6982_MOESM1_ESM.pdf]

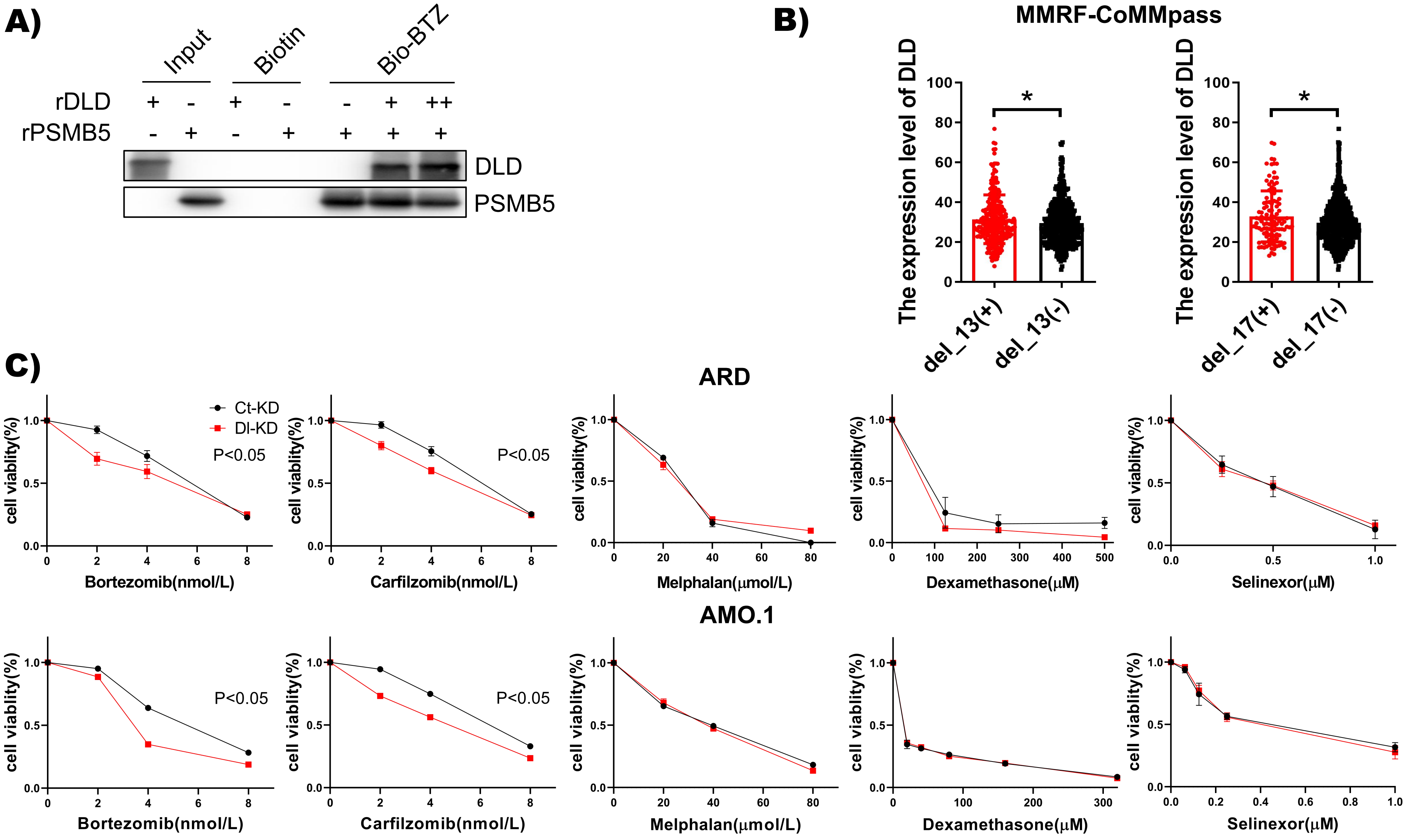

Figure S1

(A) The result showed that with the increase of DLD protein, the amount of PSMB5 combined with BTZ decreased, which indicated a competitive binding relationship between DLD and PSMB5. (B) In MMRF CoMMpass, the patients with del(13) or del(17) had higher expression of DLD ($p<0.05$). (C) The CCK-8 cell viability test results showed that after knocking down DLD, AMO.1 and ARD cells were only more sensitive to PIs (BTZ, CFZ) ($p<0.05$).

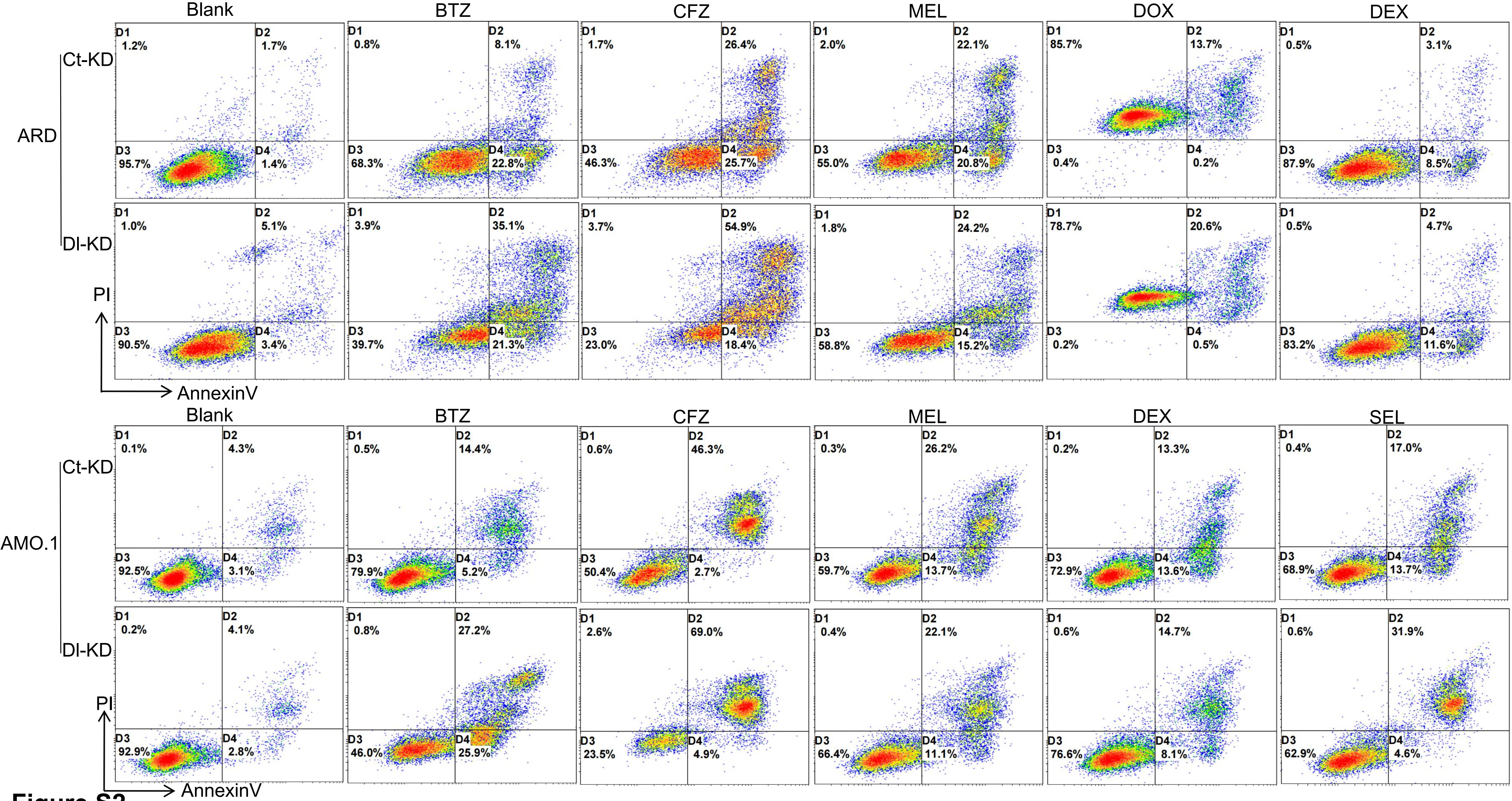

Figure S2

The results showed representative images of flow cytometry in Figure 3B.

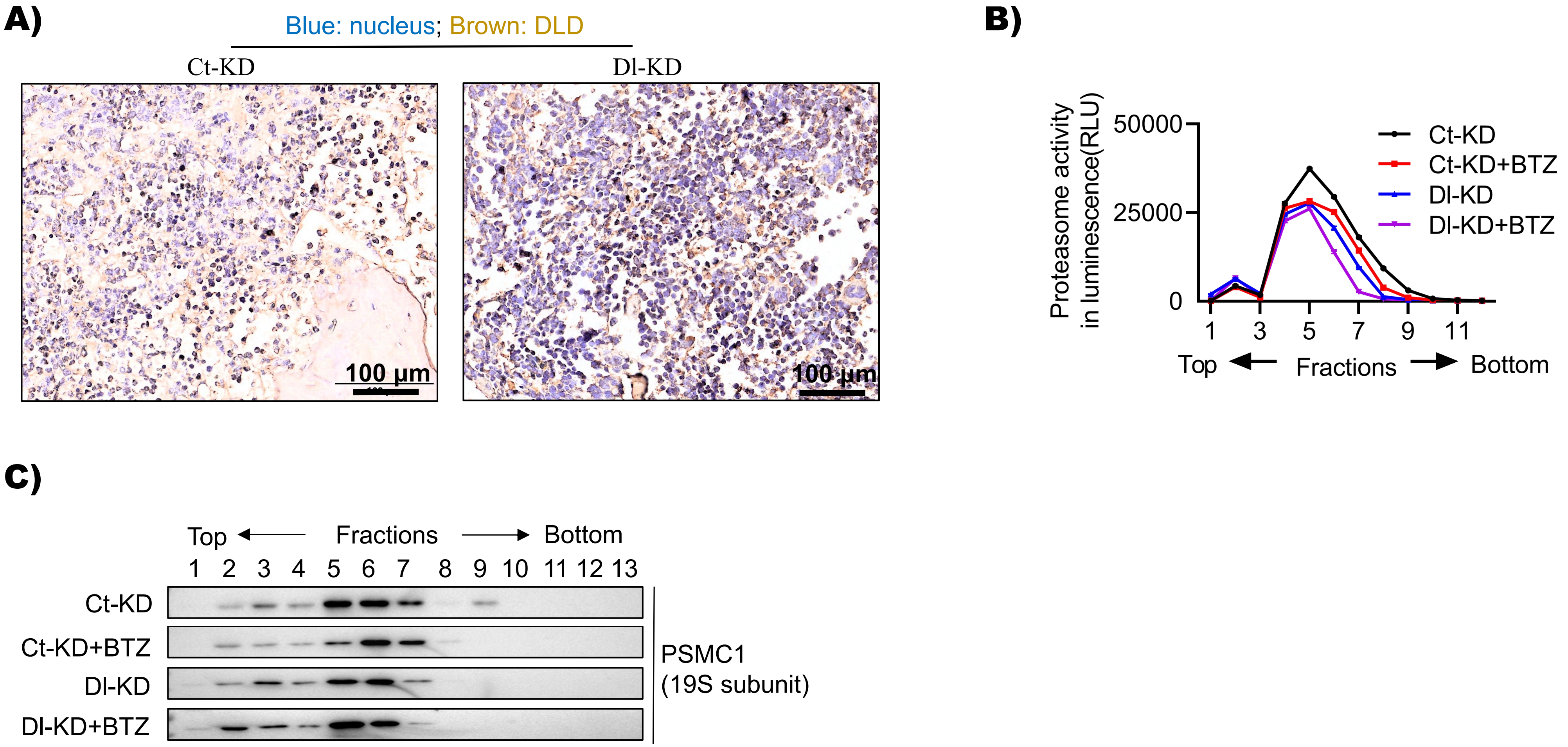

Figure S3

(A) The mouse leg bones were prepared into sections by decalcification and paraffin embedding, and immunohistochemical staining was performed to detect the expression of DLD. The IHC results confirmed the knockdown of DLD in tumor bearing mice. (B,C) The results showed that the addition of BTZ to Ct-KD or Dl-KD cells inhibited the assembly of their proteasomes, further confirming that BTZ can regulate the assembly of proteasomes by the same method as Figures 4E and F.

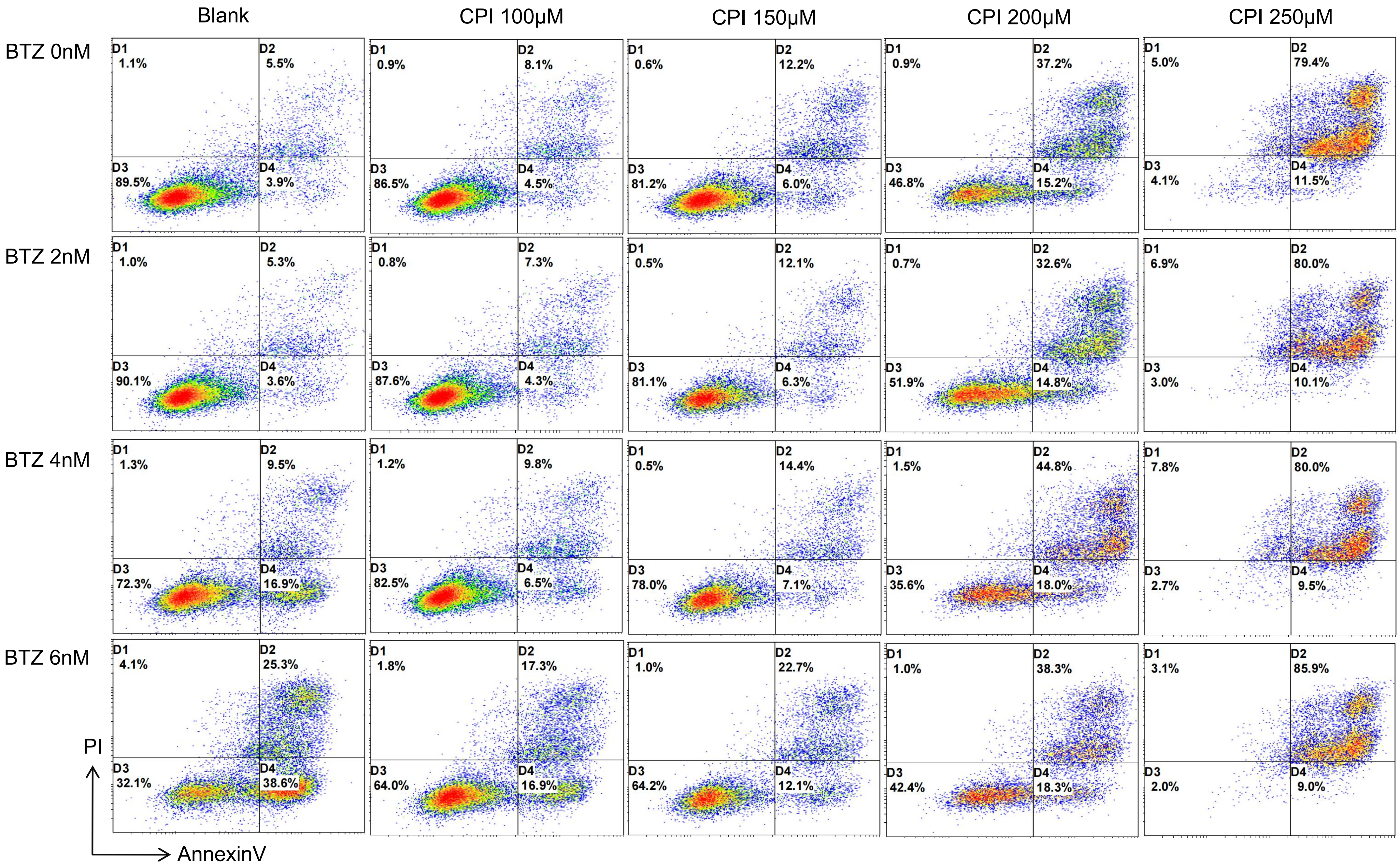

Figure S4

The results showed representative images of flow cytometry in Figure 5C.
