# Supplementary material for: Dihydrolipoamide dehydrogenase (DLD) is a novel molecular target of bortezomib
Source: Cell Death Dis. 2024 Aug 13;15(8):588. doi: 10.1038/s41419-024-06982-2 (PMC11322525; doi:10.1038/s41419-024-06982-2)
Supplement: Supplementary file 2 — Supple. Table 1 [file 41419_2024_6982_MOESM2_ESM.pdf]

| Protein names | Gene names | Intensity | Mol. weight [kDa] | Protein dots |
|---|---|---|---|---|
| Ig mu chain C region | IGHM | 61430000 | 49.439 | 1 |
| C4b-binding protein alpha chain | C4BPA | 2620200 | 67.033 | 1 |
| Semenogelin-1;Alpha-inhibin-92;Alpha-inhibin-31;Seminal basic | SEMG1 | 5589400 | 52.13 | 1 |
| Prolactin-inducible protein | PIP | 3534300 | 16.572 | 1 |
| Dihydrolipoyllysine-residue succinyltransferase component of 2-oxoglutarate dehydrogenase complex, mitochondrial | DLST | 1200800000 | 48.755 | 1 |
| Dermcidin;Survival-promoting peptide;DCD-1 | DCD | 45558000 | 11.284 | 1 |
| ATPase family AAA domain-containing protein 3C;ATPase family AAA domain-containing protein 3B | ATAD3C;ATAD3B | 578430000 | 46.379 | 1 |
| Tubulin polyglutamylase TTLL11 | TTLL11 | 545950000 | 87.611 | 1 |
| RB1-inducible coiled-coil protein 1 | RB1CC1 | 26907000 | 183.09 | 1 |
| Protein Shroom3 | SHROOM3 | 1398100000 | 216.85 | 1 |
| Tensin-1 | TNS1 | 1647300 | 185.7 | 1 |
| MAP3K12-binding inhibitory protein 1 | MBIP | 3757300 | 39.281 | 1 |
| Spermatogenesis-associated protein 7 | SPATA7 | 166550000 | 67.718 | 1 |
| Kallikrein-11;Kallikrein-11 inactive chain 1;Kallikrein-11 inactive chain | KLK11 | 208910000 | 31.059 | 1 |
| Ankyrin repeat domain-containing protein 26 | ANKRD26 | 19706000 | 196.41 | 1 |
| Pyruvate dehydrogenase protein X component, mitochondrial | PDHX | 1073100000 | 54.122 | 2 |
| MutS protein homolog 5 | MSH5 | 1024800000 | 92.874 | 2 |
| Serine/threonine-protein phosphatase 6 regulatory subunit 2 | PPP6R2 | 13025000 | 104.94 | 2 |
| Semenogelin-1;Alpha-inhibin-92;Alpha-inhibin-31;Seminal basic | SEMG1 | 3519700 | 52.13 | 2 |
| Propionyl-CoA carboxylase beta chain, mitochondrial | PCCB | 3153000 | 58.215 | 2 |
| Prolactin-inducible protein | PIP | 3021200 | 16.572 | 2 |
| Dermcidin;Survival-promoting peptide;DCD-1 | DCD | 98248000 | 11.284 | 2 |
| ATPase family AAA domain-containing protein 3C;ATPase family AAA domain-containing protein 3B | ATAD3C;ATAD3B | 273670000 | 46.379 | 2 |
| Tubulin polyglutamylase TTLL11 | TTLL11 | 397390000 | 87.611 | 2 |
| RB1-inducible coiled-coil protein 1 | RB1CC1 | 31366000 | 183.09 | 2 |
| Protein Shroom3 | SHROOM3 | 2214400000 | 216.85 | 2 |
| Spermatogenesis-associated protein 7 | SPATA7 | 139040000 | 67.718 | 2 |
| Kallikrein-11;Kallikrein-11 inactive chain 1;Kallikrein-11 inactive chain | KLK11 | 244060000 | 31.059 | 2 |
| MutS protein homolog 5 | MSH5 | 931200000 | 92.874 | 3 |
| Ig mu chain C region | IGHM | 3980000 | 49.439 | 3 |
| Semenogelin-1;Alpha-inhibin-92;Alpha-inhibin-31;Seminal basic | SEMG1 | 5648100 | 52.13 | 3 |
| Lipoamide acyltransferase component of branched-chain alpha-keto acid dehydrogenase complex, mitochondrial | DBT | 213700000 | 53.486 | 3 |

| | | | | |
|---|---|---|---|---|
| ATPase family AAA domain-containing protein 3C;ATPase family AAA domain-containing protein 3B | ATAD3C;ATAD3B | 224330000 | 46.379 | 3 |
| Tubulin polyglutamylase TTLL11 | TTLL11 | 390040000 | 87.611 | 3 |
| RB1-inducible coiled-coil protein 1 | RB1CC1 | 100610000 | 183.09 | 3 |
| Protein Shroom3 | SHROOM3 | 1302900000 | 216.85 | 3 |
| Spermatogenesis-associated protein 7 | SPATA7 | 180440000 | 67.718 | 3 |
| Kallikrein-11;Kallikrein-11 inactive chain 1;Kallikrein-11 inactive chain | KLK11 | 349490000 | 31.059 | 3 |
| Semenogelin-1;Alpha-inhibin-92;Alpha-inhibin-31;Seminal basic | SEMG1 | 6448900 | 52.13 | 4 |
| Lipoamide acyltransferase component of branched-chain alpha-keto acid dehydrogenase complex, mitochondrial | DBT | 12074000 | 53.486 | 4 |
| Dystrophin | DMD | 62447000 | 426.74 | 4 |
| Prolactin-inducible protein | PIP | 1981100 | 16.572 | 4 |
| Collagen alpha-5(IV) chain | COL4A5 | 3657600 | 161.04 | 4 |
| Dihydrolipoyllysine-residue succinyltransferase component of 2-oxoglutarate dehydrogenase complex, mitochondrial | DLST | 4543600 | 48.755 | 4 |
| ATPase family AAA domain-containing protein 3C;ATPase family AAA domain-containing protein 3B | ATAD3C;ATAD3B | 262270000 | 46.379 | 4 |
| Unconventional myosin-XVIIIb | MYO18B | 12083000 | 285.21 | 4 |
| RB1-inducible coiled-coil protein 1 | RB1CC1 | 105600000 | 183.09 | 4 |
| Spermatogenesis-associated protein 7 | SPATA7 | 181240000 | 67.718 | 4 |
| Kallikrein-11;Kallikrein-11 inactive chain 1;Kallikrein-11 inactive chain | KLK11 | 166030000 | 31.059 | 4 |
| Serine/threonine-protein phosphatase 6 regulatory subunit 2 | PPP6R2 | 12093000 | 104.94 | 5 |
| Ig mu chain C region | IGHM | 4298900 | 49.439 | 5 |
| Keratin, type II cytoskeletal 6B | KRT6B | 18507000 | 60.066 | 5 |
| Semenogelin-1;Alpha-inhibin-92;Alpha-inhibin-31;Seminal basic | SEMG1 | 18234000 | 52.13 | 5 |
| Propionyl-CoA carboxylase beta chain, mitochondrial | PCCB | 634110000 | 58.215 | 5 |
| Dystrophin | DMD | 61933000 | 426.74 | 5 |
| Prolactin-inducible protein | PIP | 2394500 | 16.572 | 5 |
| C4b-binding protein beta chain | C4BPB | 307860 | 28.357 | 5 |
| Dermcidin;Survival-promoting peptide;DCD-1 | DCD | 566930000 | 11.284 | 5 |
| ATPase family AAA domain-containing protein 3C;ATPase family AAA domain-containing protein 3B | ATAD3C;ATAD3B | 72825000 | 46.379 | 5 |
| Tubulin polyglutamylase TTLL11 | TTLL11 | 327000000 | 87.611 | 5 |
| Protein Shroom3 | SHROOM3 | 1152300000 | 216.85 | 5 |
| MAP3K12-binding inhibitory protein 1 | MBIP | 3309700 | 39.281 | 5 |
| Spermatogenesis-associated protein 7 | SPATA7 | 154650000 | 67.718 | 5 |
| Kallikrein-11;Kallikrein-11 inactive chain 1;Kallikrein-11 inactive chain | KLK11 | 313280000 | 31.059 | 5 |
| Semenogelin-1;Alpha-inhibin-92;Alpha-inhibin-31;Seminal basic | SEMG1 | 139840000 | 52.13 | 6 |
| Apolipoprotein D | APOD | 87114000 | 21.275 | 6 |

| | | | | |
|---|---|---|---|---|
| Dermcidin;Survival-promoting peptide;DCD-1 | DCD | 213960000 | 11.284 | 6 |
| Semenogelin-2 | SEMG2 | 7536000 | 65.444 | 6 |
| ATPase family AAA domain-containing protein 3C;ATPase family AAA domain-containing protein 3B | ATAD3C;ATAD3B | 473620000 | 46.379 | 6 |
| Tubulin polyglutamylase TTLL11 | TTLL11 | 29341000 | 87.611 | 6 |
| RB1-inducible coiled-coil protein 1 | RB1CC1 | 28405000 | 183.09 | 6 |
| Protein Shroom3 | SHROOM3 | 1195800000 | 216.85 | 6 |
| Methylcrotonoyl-CoA carboxylase beta chain, mitochondrial | MCCC2 | 136800000 | 61.332 | 6 |
| Spermatogenesis-associated protein 7 | SPATA7 | 155320000 | 67.718 | 6 |
| Kallikrein-11;Kallikrein-11 inactive chain 1;Kallikrein-11 inactive chain | KLK11 | 193520000 | 31.059 | 6 |
| Semenogelin-1;Alpha-inhibin-92;Alpha-inhibin-31;Seminal basic | SEMG1 | 257770000 | 52.13 | 7 |
| Zinc-alpha-2-glycoprotein | AZGP1 | 3897000 | 34.258 | 7 |
| Dermcidin;Survival-promoting peptide;DCD-1 | DCD | 9969700 | 11.284 | 7 |
| Semenogelin-2 | SEMG2 | 333800000 | 65.444 | 7 |
| Desmocollin-1 | DSC1 | 2942200 | 99.986 | 7 |
| ATPase family AAA domain-containing protein 3C;ATPase family AAA domain-containing protein 3B | ATAD3C;ATAD3B | 167770000 | 46.379 | 7 |
| Thioredoxin domain-containing | TXNDC11 | 1120900 | 110.53 | 7 |
| Tubulin polyglutamylase TTLL11 | TTLL11 | 799840000 | 87.611 | 7 |
| RB1-inducible coiled-coil protein 1 | RB1CC1 | 56912000 | 183.09 | 7 |
| Methylcrotonoyl-CoA carboxylase beta chain, mitochondrial | MCCC2 | 2994800000 | 61.332 | 7 |
| Calmodulin-like protein 5 | CALML5 | 12026000 | 15.892 | 7 |
| Spermatogenesis-associated protein 7 | SPATA7 | 133130000 | 67.718 | 7 |
| Kallikrein-11;Kallikrein-11 inactive chain 1;Kallikrein-11 inactive chain | KLK11 | 801850000 | 31.059 | 7 |
| Ig kappa chain C region | IGKC | 4720000 | 11.765 | 8 |
| Ig mu chain C region | IGHM | 14186000 | 49.439 | 8 |
| Keratin, type II cytoskeletal 6B | KRT6B | 23906000 | 60.066 | 8 |
| Semenogelin-1;Alpha-inhibin-92;Alpha-inhibin-31;Seminal basic | SEMG1 | 25613000 | 52.13 | 8 |
| Dihydrolipoyl dehydrogenase, mitochondrial | DLD | 14711000 | 54.177 | 8 |
| Prolactin-inducible protein | PIP | 2093500 | 16.572 | 8 |
| Collagen alpha-5(IV) chain | COL4A5 | 10012000 | 161.04 | 8 |
| ATPase family AAA domain-containing protein 3C;ATPase family AAA domain-containing protein 3B | ATAD3C;ATAD3B | 425800000 | 46.379 | 8 |
| Unconventional myosin-XVIIIb | MYO18B | 3310500 | 285.21 | 8 |
| Tubulin polyglutamylase TTLL11 | TTLL11 | 307980000 | 87.611 | 8 |
| RB1-inducible coiled-coil protein 1 | RB1CC1 | 108120000 | 183.09 | 8 |
| Tensin-1 | TNS1 | 10128000 | 185.7 | 8 |
| Spermatogenesis-associated protein 7 | SPATA7 | 116340000 | 67.718 | 8 |
| Kallikrein-11;Kallikrein-11 inactive chain 1;Kallikrein-11 inactive chain | KLK11 | 263620000 | 31.059 | 8 |
| Semenogelin-1;Alpha-inhibin-92;Alpha-inhibin-31;Seminal basic | SEMG1 | 13531000 | 52.13 | 9 |

| | | | | |
|---|---|---|---|---|
| Prolactin-inducible protein | PIP | 1461300 | 16.572 | 9 |
| ATPase family AAA domain-containing protein 3C;ATPase family AAA domain-containing protein 3B | ATAD3C;ATAD3B | 186120000 | 46.379 | 9 |
| RB1-inducible coiled-coil protein 1 | RB1CC1 | 118110000 | 183.09 | 9 |
| Protein Shroom3 | SHROOM3 | 1324300000 | 216.85 | 9 |
| Spermatogenesis-associated protein 7 | SPATA7 | 156380000 | 67.718 | 9 |
| Kallikrein-11;Kallikrein-11 inactive chain 1;Kallikrein-11 inactive chain | KLK11 | 230590000 | 31.059 | 9 |
| tRNA-splicing ligase RtcB homolog | RTCB | 21953000 | 55.21 | 9 |
| Serine/threonine-protein phosphatase 6 regulatory subunit 2 | PPP6R2 | 4996500 | 104.94 | 10 |
| Serine/threonine-protein kinase OSR1 | OXSR1 | 2481800 | 58.022 | 10 |
| Keratin, type II cytoskeletal 6B | KRT6B | 5693900 | 60.066 | 10 |
| Semenogelin-1;Alpha-inhibin-92;Alpha-inhibin-31;Seminal basic | SEMG1 | 60482000 | 52.13 | 10 |
| Pyruvate dehydrogenase E1 component subunit beta, mitochondrial | PDHB | 43472000 | 39.233 | 10 |
| ATPase family AAA domain-containing protein 3C;ATPase family AAA domain-containing protein 3B | ATAD3C;ATAD3B | 21617000 | 46.379 | 10 |
| RB1-inducible coiled-coil protein 1 | RB1CC1 | 85886000 | 183.09 | 10 |
| Spermatogenesis-associated protein 7 | SPATA7 | 101030000 | 67.718 | 10 |
| Kallikrein-11;Kallikrein-11 inactive chain 1;Kallikrein-11 inactive chain | KLK11 | 197960000 | 31.059 | 10 |
