# Supplementary figures and images for: Dihydrolipoamide dehydrogenase (DLD) is a novel molecular target of bortezomib
Source: Cell Death Dis. 2024 Aug 13;15(8):588. doi: 10.1038/s41419-024-06982-2 (PMC11322525; doi:10.1038/s41419-024-06982-2)

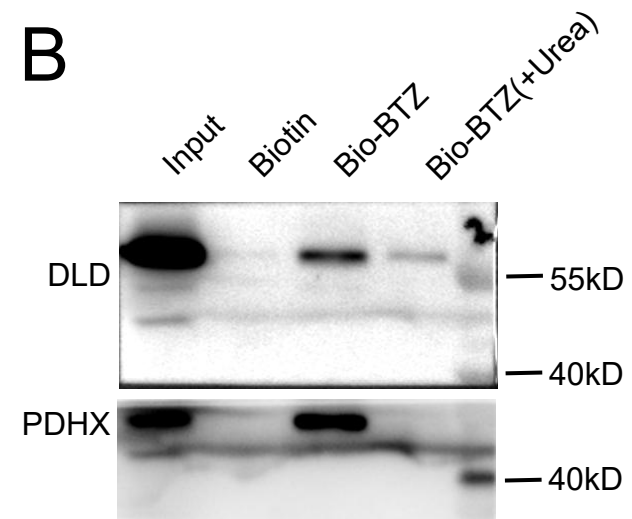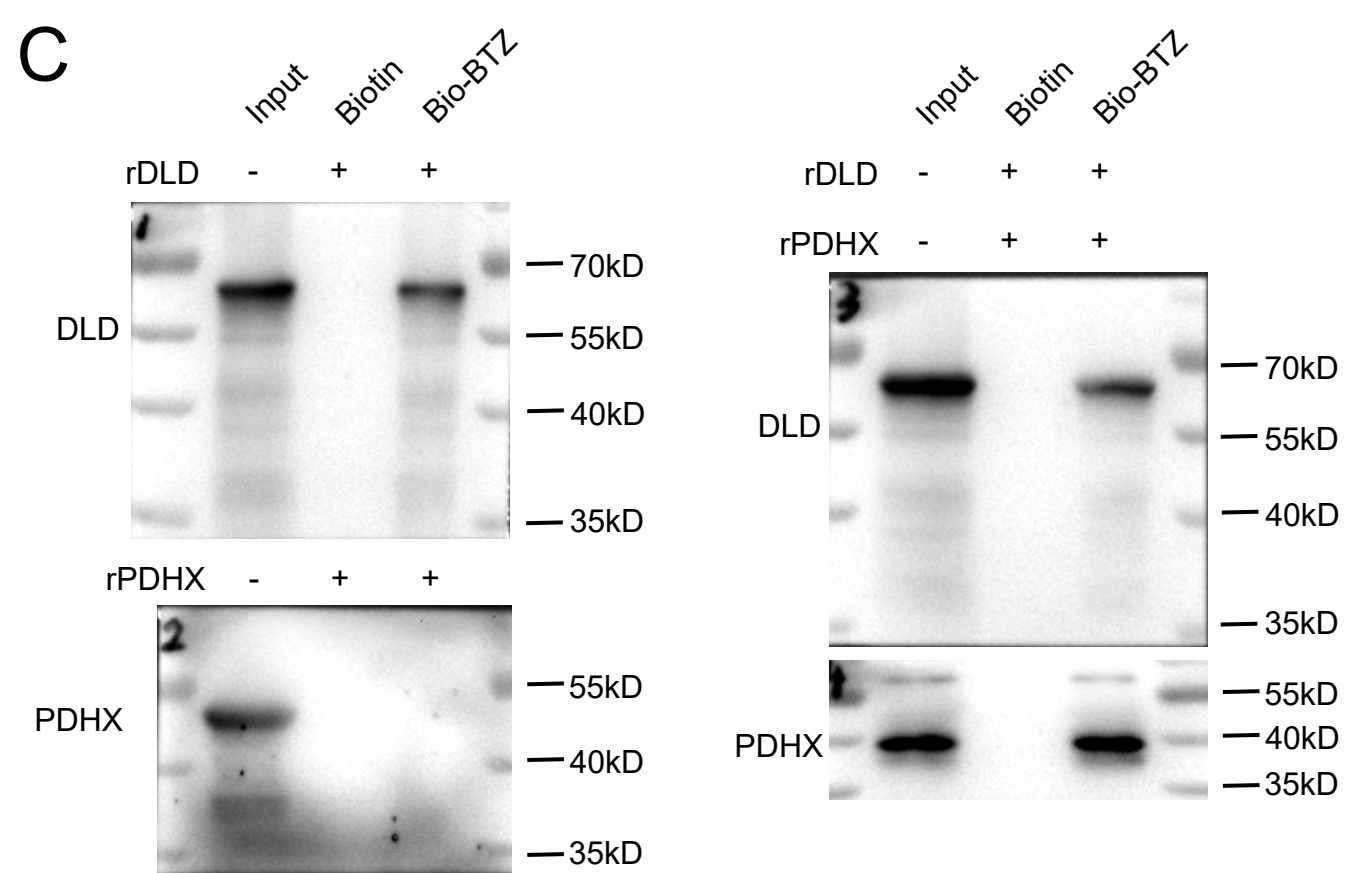

Figure 1

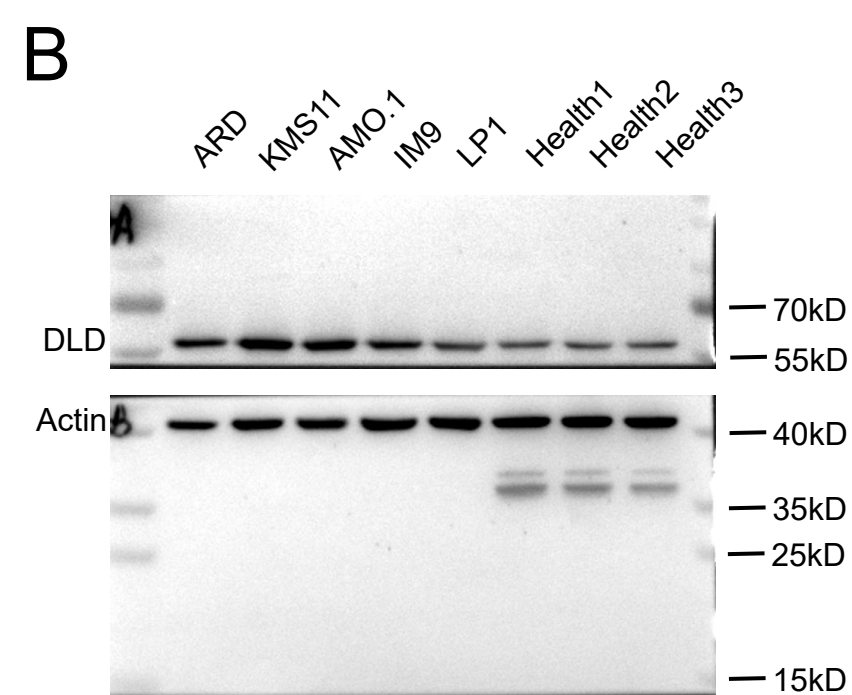

Figure 2

A

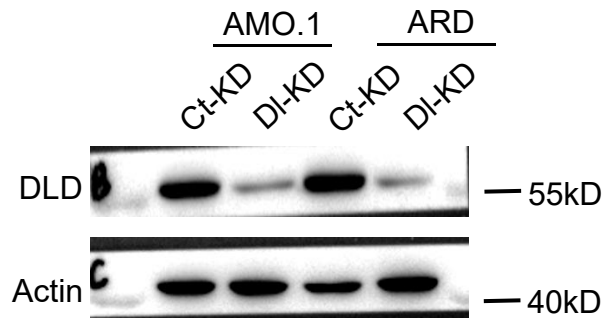

C

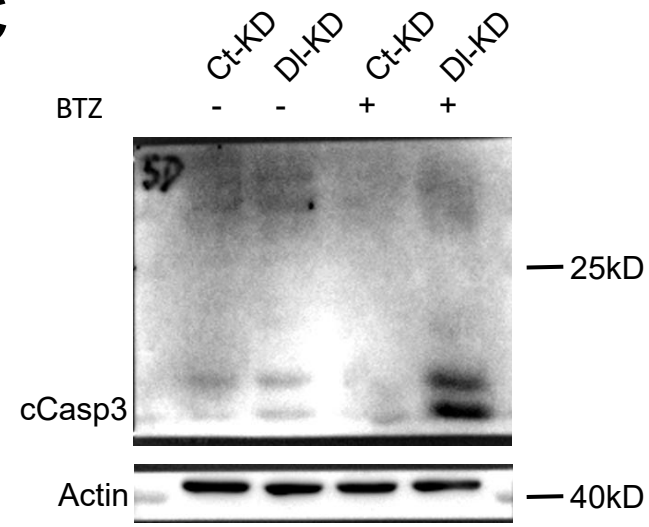

Figure 3

A

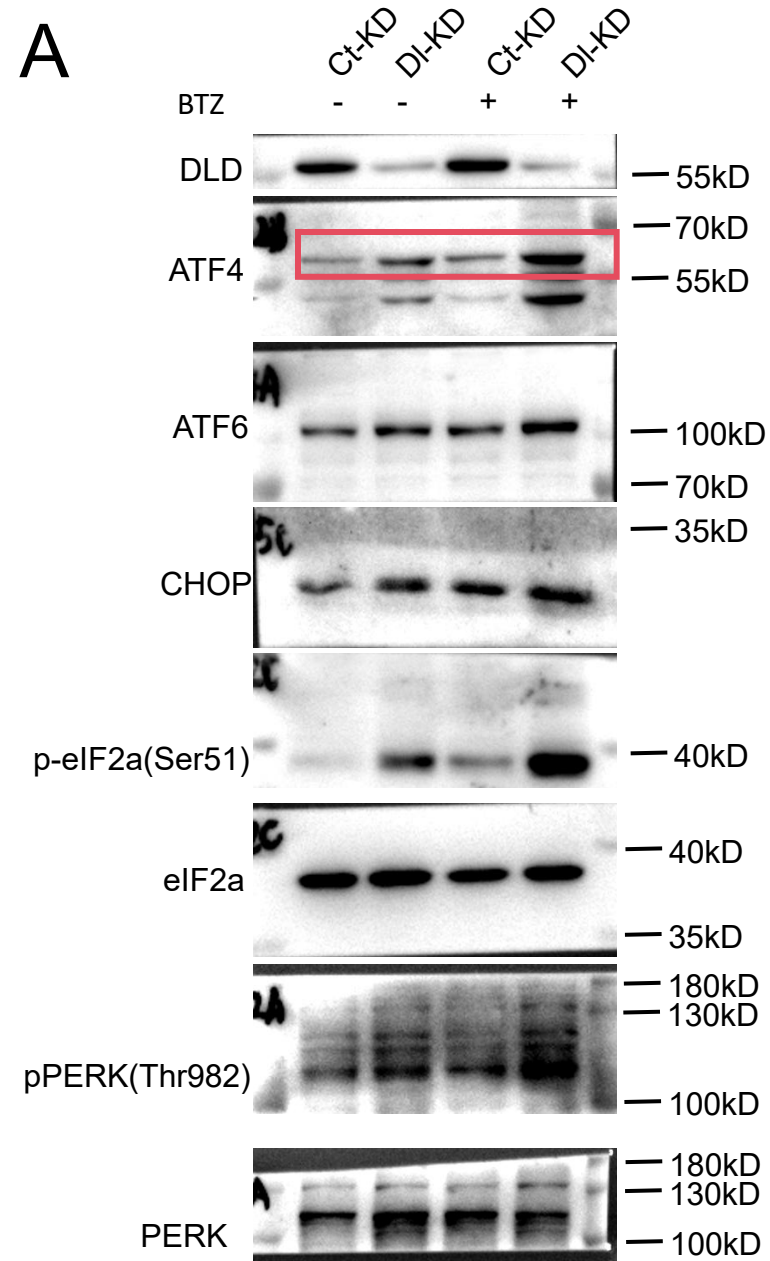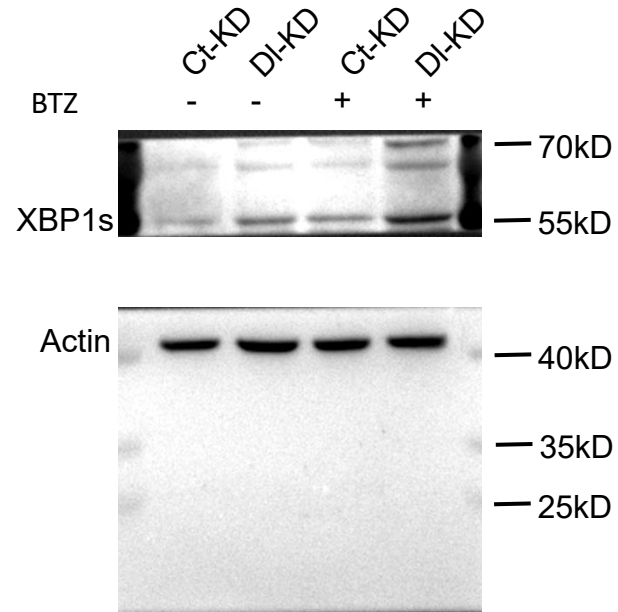

C

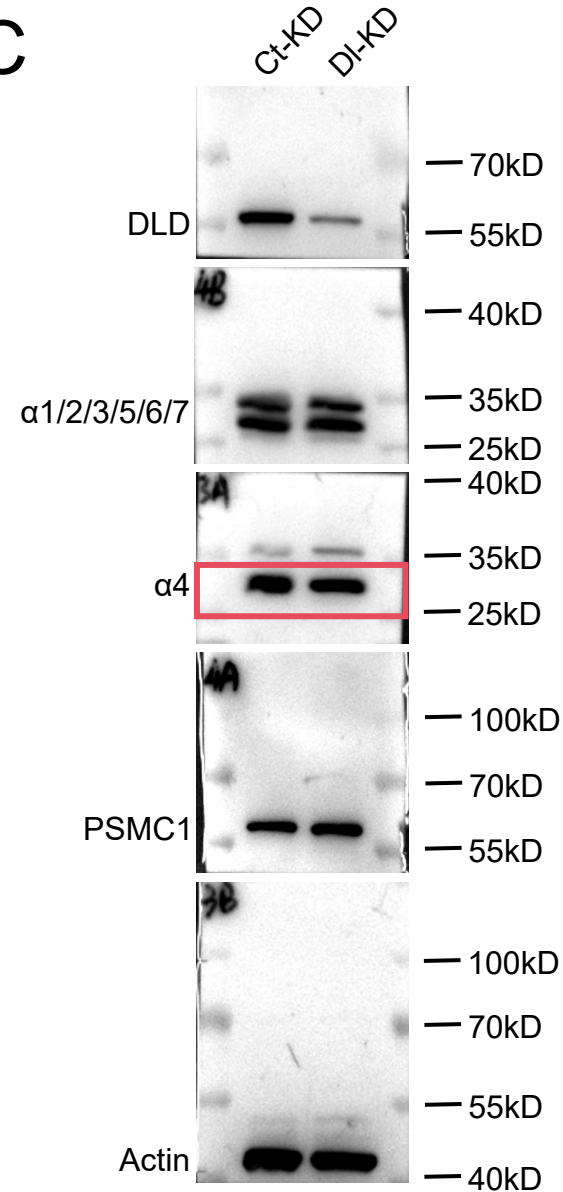

Figure 4

E

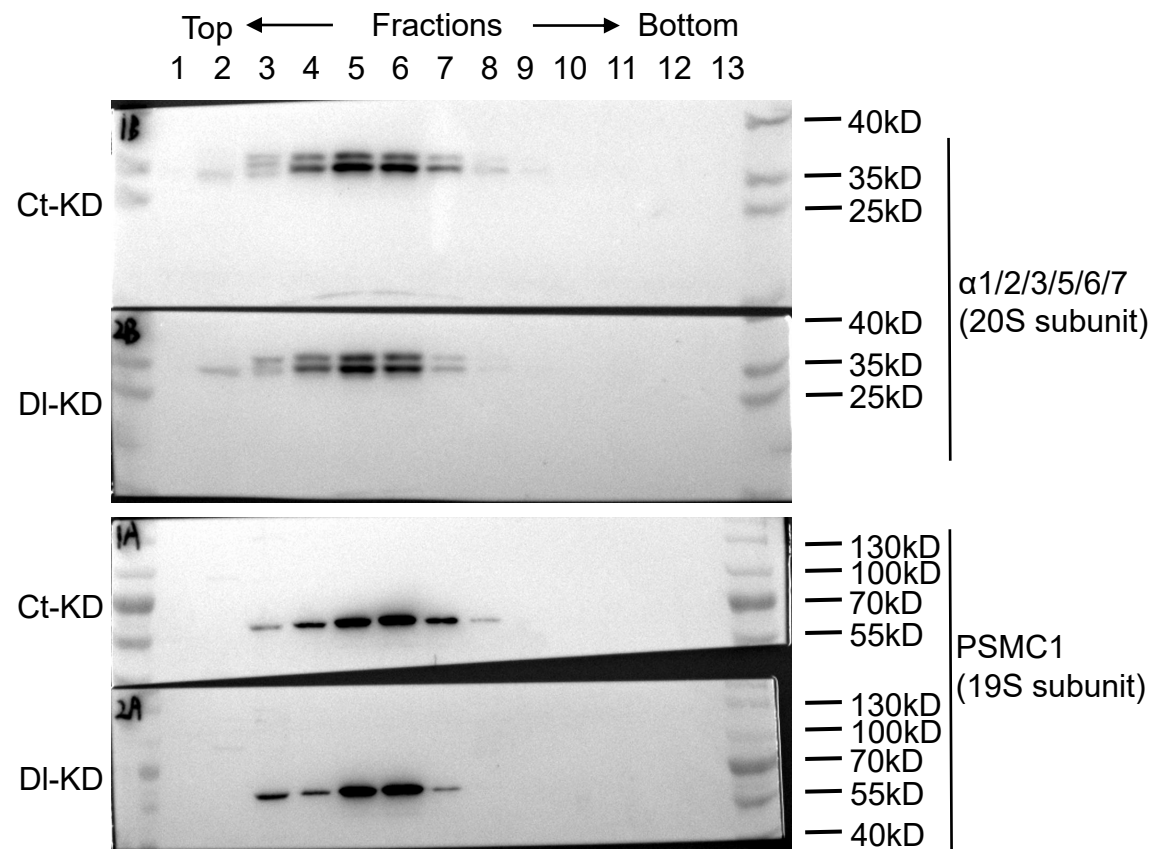

J

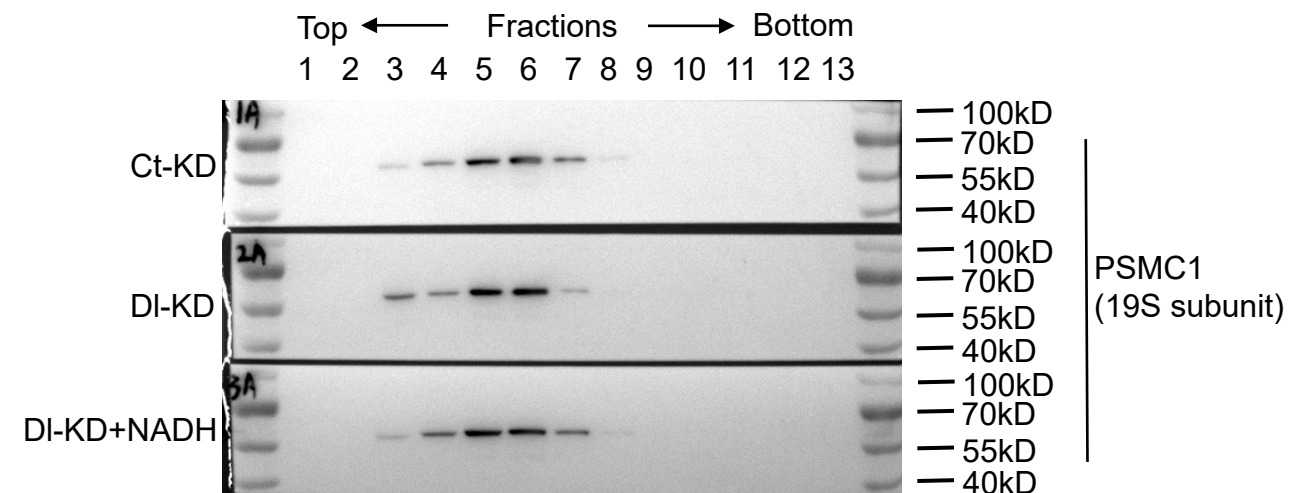

Figure 4

A

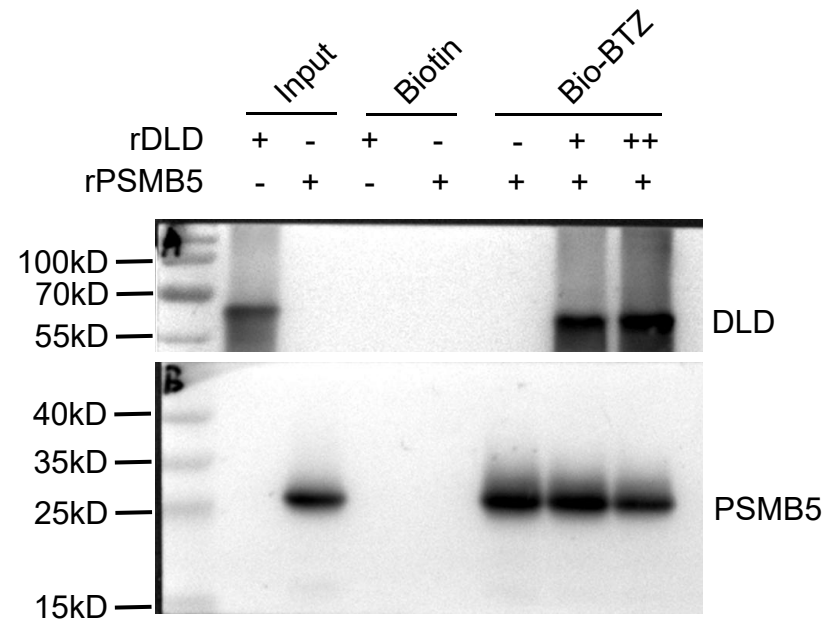

Supplementary FigureS1

C

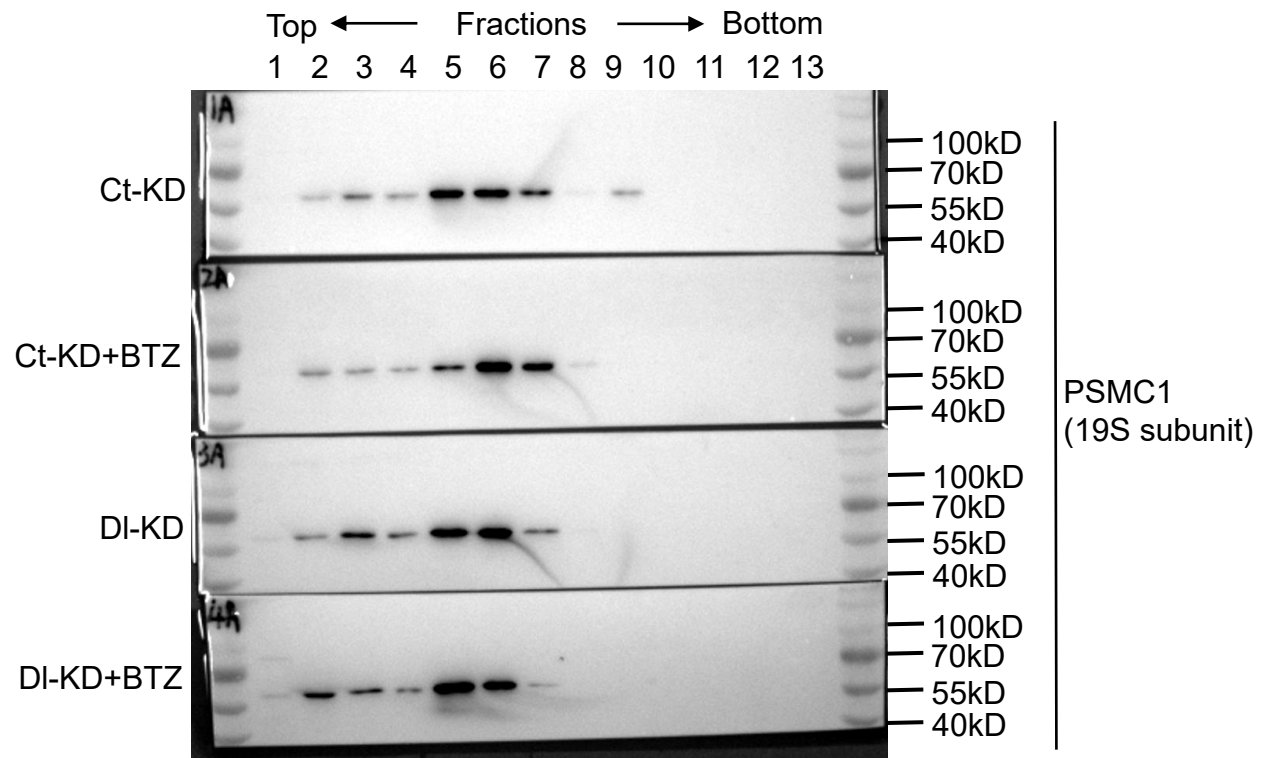

Supplementary FigureS3

Supplement: Supplementary file 3 — Supplemental material [file 41419_2024_6982_MOESM3_ESM.pdf]
